# Deciphering the role of tRNA-derived fragments in neurological and psychiatric disease pathogenesis

**DOI:** 10.3389/fncel.2025.1663788

**Published:** 2025-12-03

**Authors:** Huseyin Kocakusak, Aysu Başak Kök, Bilgesu Ozturk, Bilge Karacicek, Sermin Genc

**Affiliations:** 1 Izmir Biomedicine and Genome Center, Izmir, Türkiye; 2 Izmir International Biomedicine and Genome Institute, Dokuz Eylul University, Izmir, Türkiye; 3 Department of Neuroscience, Institute of Health Sciences, Dokuz Eylul University, Izmir, Türkiye

**Keywords:** tsRNA, tRFs, neurological diseases, biomarkers, SncRNAs

## Abstract

tRNA-derived small RNAs (tsRNAs) have recently gained attention as important regulatory non-coding RNAs (ncRNAs). Among these, tRNA-derived fragments (tRFs) constitute a distinct and well-defined subset. These small molecules play essential roles in maintaining cellular homeostasis and have been increasingly implicated in disease pathogenesis. This comprehensive review specifically concentrates on tRFs, takes a closer look at their diverse mechanisms of action and their impact on key cellular processes. Specific focus is placed on their functions within the central nervous system (CNS) and their involvement in the molecular pathways driving neurological diseases and neurodevelopmental disorders. Besides their pathological roles, the review covers fundamental aspects of tRFs, including their biogenesis, classification, and structural features. It also describes latest methods for tRFs detection, prediction, and validation. Overall, the review points out the ongoing need for research in this area, especially when it comes to applying these findings clinically. Importantly, it highlights their potential as useful biomarkers and even targets for treatment in neurological diseases.

## Graphical Abstract

**Figure:**
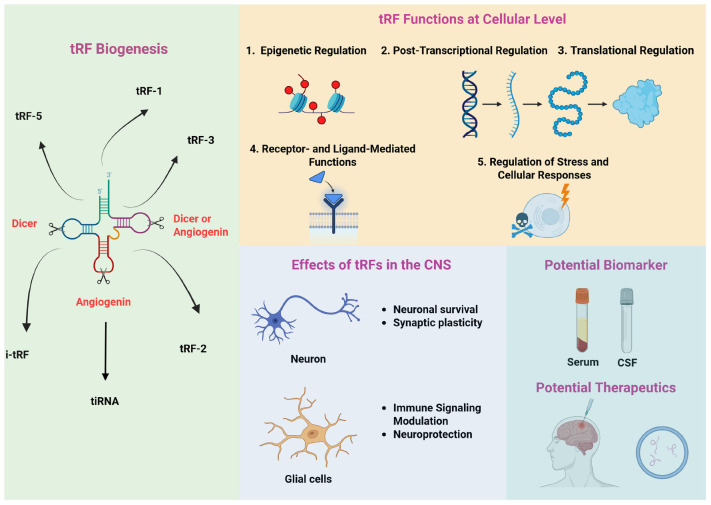
Overview of key tRFs: their biogenesis, functions, roles in the CNS, and biomarker/therapeutic potential. The left panel summarizes the enzymatic cleavages that generate distinct tRF subtypes during tRF biogenesis. The upper panel highlights tRF functions at the cellular level, including epigenetic, post-translational, and translational regulation, their roles in stress responses, and receptor- and ligand-mediated modulation. The lower left panel depicts the involvement of tRFs in key CNS processes. Finally, bottom right panel illustrates the potential of tRFs as biomarkers in different sample sources (e.g., serum, CSF) and their possible therapeutic applications for neurological and psychiatric disorders.

## 1 Introduction

### 1.1 Discovery of tsRNA

Transfer RNAs (tRNAs) are type of non-coding RNAs (ncRNAs) which acts as an adapter molecule that facilitates protein synthesis by helping the ribosome interpret nucleotide triplets. It links the genetic information on messenger RNA (mRNA) to the corresponding amino acid sequences (Anderson and Ivanov, 2014). Apart from its well-established function, tRNAs are the source for the production of tRNA-derived small RNAs (tsRNAs) (Su et al., 2020b). tsRNAs had emerged from studies in *Escherichia coli* (*E. Coli*). Researchers observed that upon bacteriophage invasion, *E. Coli* cells generated short RNA fragments from tRNA as part of their cellular response. This discovery provided an important evidence that tRFs generation represents a regulated cellular event rather than random degradation or turnover (Levitz et al., 1990; Li and Stanton, 2021). A breakthrough in understanding tRFs occurred in 2009 when Lee et al. (2009) investigated these novel small RNAs (sRNA). By extracting 17–26 nucleotide fragments from prostate cancer cell lines (LNCaP and C4-2) and employing 454 deep sequencing with specialized analytical pipelines, they identified 17 tsRNAs originating from either precursor tRNAs or the processing of mature tRNAs’ 5′ or 3′ regions (Lee et al., 2009). Concurrent work by Cole et al. (2009) team independently confirmed the existence of abundant sRNA molecules derived specifically from mature tRNA 5′ ends when they sequenced RNA from HeLa cell nucleoli. Subsequent investigations have indicated that tsRNA biogenesis occurs through precise cleavage at specific sites on tRNA molecules. Importantly, both the production mechanisms and the profile of resulting fragment changes depending on the cell type and physiological conditions. This controlled mechanism yields distinct tsRNA classes that perform specific functions within cells (Anderson and Ivanov, 2014; Soares and Santos, 2017; Kuhle et al., 2023). The integration of experimental techniques, high-throughput small RNA sequencing, and bioinformatics analysis has made the detection, identification, and quantification of tsRNA easy. This enabled researchers to study biological roles of tsRNA’s across diverse samples and clinical contexts (Yu et al., 2024).

### 1.2 Biogenesis, classification and structure of tsRNAs

tRNA-derived small RNAs classification system is based on which structural region of the parent tRNA molecule was cleaved to produce the fragment, the length of tsRNAs and the conditions under which they are produced (Lee et al., 2009; Kumar et al., 2015; George et al., 2022). According to this system, there are two main classes: tRNA-derived stress-induced RNAs (tiRNAs or tRNA halves) and tRFs. tiRNAs contain some double-stranded structural elements and generally 30–40 nucleotides in length. The ribonuclease angiogenin (ANG) cleaves tRNAs at the anticodon loop. This precise endonucleolytic cleavage results in the production of two distinct fragments. These fragments are named as 5′ tiRNAs and 3′ tiRNAs depending on whether they contain the 5′ or the 3′ end of the parent tRNA, respectively (Fu et al., 2009). However, tRFs have also been found to be produced independently from ANG under specific cellular conditions (Rashad et al., 2021). Ultraviolet (UV) radiation, heat shock, oxidative stress, viral infection, nutrient deprivation, or hypoxia have been found to induce tiRNAs production. These indicate that tiRNA production is linked to stress conditions (Thompson et al., 2008; Fu et al., 2009; Yamasaki et al., 2009; Saikia and Hatzoglou, 2015; Pan et al., 2021; Ajmeriya et al., 2024). tRFs are generated through processing of tRNA precursors (pre-tRNAs) or the endonucleolytic cleavage of mature tRNAs. The specific location where these cleavages occur on the tRNA molecule determines the type of tsRNA that will be produced. There are six main types of tRFs in eukaryotes: tRF-1 (3′ U-tRF), 5′ U-tRFs, tRF-2, tRF-3 (3′ tRFs), tRF-5 (5′ tRFs), and i-tRF. The nomenclature of tRFs varies in the literature as researchers often use alternative classification systems and terminology to describe the same species.

tRF-1s are generated through the cleavage of 3’ untranslated region (3′ UTR) of pre-tRNA by ELAC2/RNaseZ, their length is variable and shows a broad length distribution. Because they carry a poly U sequence, tRF-1s are also named as 3′ U-tRF (Karousi et al., 2019; Yang et al., 2023). On the other hand, 5′ U-tRFs are generated from the 5′ leader sequence of pre-tRNA and are typically 17 nucleotides in length (Weng et al., 2022). tRF-2s are fragments derived from the anticodon stem and loop region of tRNAs under hypoxic conditions. They typically do not include the 5′ or 3′ ends of pre- or mature tRNAs. Several specific tRNA species, including tRNA*^Glu^*, tRNA*^Asp^*, tRNA*^Gly^*, and tRNA*^Tyr^* have been shown to be sources of these tRFs. tRF-2s have been found to exhibit tumor-suppressive properties through their interaction with Y-box binding protein 1 (YBX1), an RNA-binding protein that normally stabilizes oncogenic transcripts. By binding to YBX1, tRF-2s effectively sequester this protein, preventing it from stabilizing cancer-promoting transcripts and thereby inhibiting tumor progression (Goodarzi et al., 2015; Kumar et al., 2016). tRF-3s are derived from the 3′ end of the mature tRNAs through specific endonucleolytic cleavage. They are generally 18–22 nucleotides in length and include the “CCA” trinucleotide at their 3′ end. tRF-3s are cleaved at the TΨC loop and this cleavage can be mediated by different enzymes depending on the cellular context, including ANG, Dicer, or other cellular exonucleases. Based on their precise cleavage sites, tRF-3s are further classified into two subtypes: tRF-3a and tRF-3b. tRF-5s are produced from the 5′ end of the mature tRNAs through Dicer mediated digestion. These cleavages occur around the D-loop or the stem region between the D-loop and anticodon loop. Depending on the precise location of the cut, different tRF-5 subtypes with varying lengths are produced: tRF-5a (14–16 nucleotides) originating from the D-loop, tRF-5b (22–24 nucleotides) from the D-stem, and tRF-5c (28–30 nucleotides) from the anticodon stem (Kumar et al., 2016; Xie et al., 2020). i-tRFs are derived from internal positions of the mature tRNA and can be produced any location and they do not contain 5′ or 3′ ends of mature tRNA (Kuscu et al., 2018). Based on cleavage sites i-tRFs are divided into three categories: A-tRF, V-tRF, and D-tRF. A-tRFs result from cleavage in the anticodon loop, V-tRFs originate from cuts in the variable region, and D-tRFs are produced by cleavage within the D loop (Chu et al., 2022).

In 2014, Kumar et al. (2014) demonstrated that the distribution of tRF-1, tRF-3, and tRF-5 are not equal across the mouse and human cell lines. Their study also showed that the expression of these transfer RNA fragments is tissue specific. Generally, tRF-1s show lower abundance compared to tRF-5 and tRF-3s in the mouse tissues and human cell lines they examined. tRF-2s and i-tRFs are found to be not as abundant as other three types of tRFs. The overall order of abundance from highest to lowest is: tRF-5, tRF-3, and tRF-1 (Kumar et al., 2014). Meta-analysis of sRNA data in HeLa cells has indicated distinct subcellular localization patterns: tRF-3s and tRF-1s are predominantly found in the cytoplasm, while tRF-5s are mainly present in the nucleus. However, these tRFs can relocate from the cytoplasm to the nucleus under certain conditions (Kumar et al., 2016).

## 2 Functions of tRFs

**FIGURE 1:**
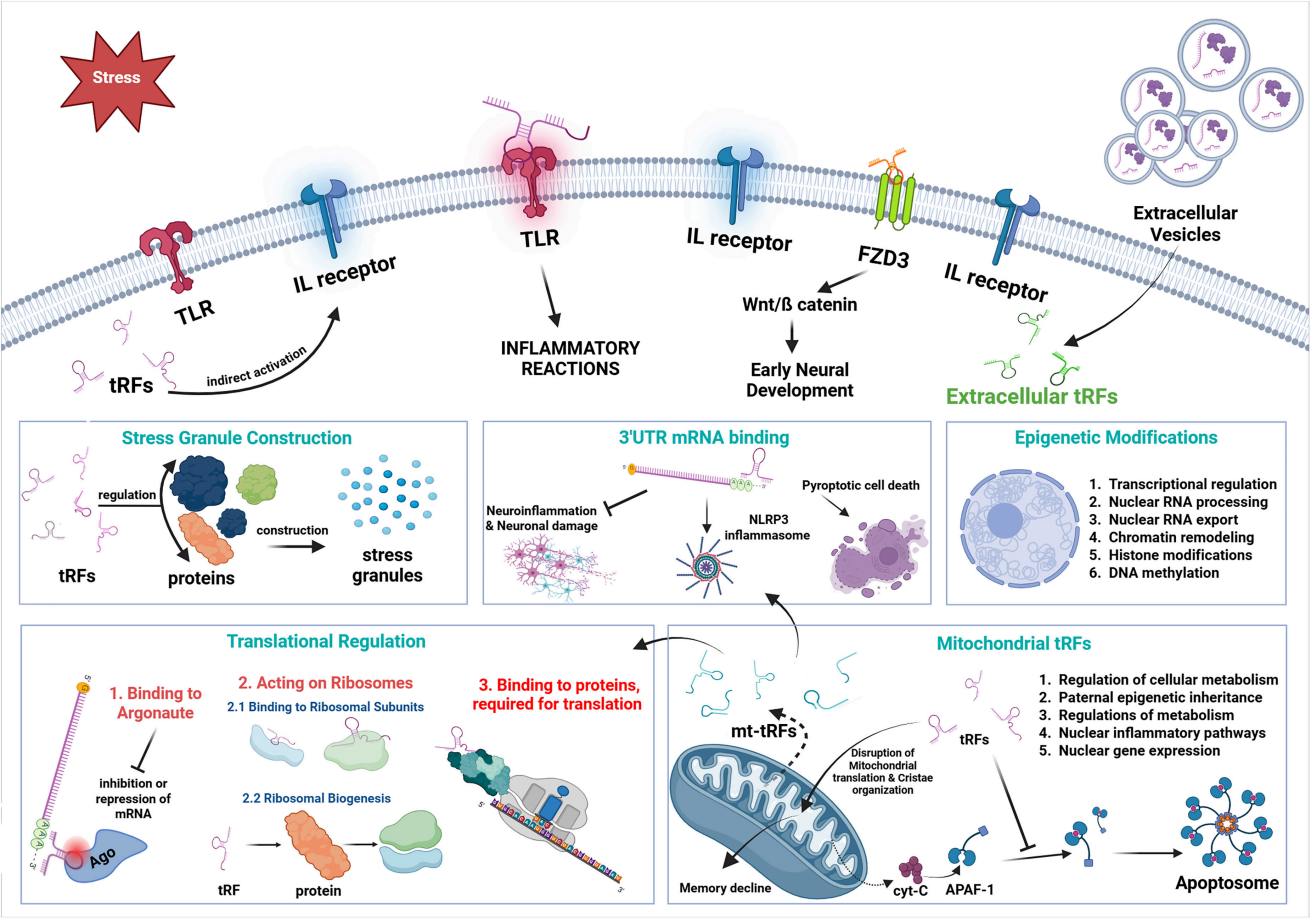
Action mechanisms of tRFs. Diagram depicts the complex regulatory processes of tRFs and mt-tRFs on neuroinflammation, translational control and neuronal homeostasis.

tRNA-derived fragments have emerged as critical regulators of gene expression, cellular stress responses, and neurodegenerative processes (Figure 1). Recent studies have revealed that these RNAs play significant roles in neuronal homeostasis and are implicated in the pathophysiology of various neurodegenerative diseases (NDs) such as Alzheimer’s disease (AD), Parkinson’s disease (PD), and Amyotrophic Lateral Sclerosis (ALS) by interacting with gene expression, translation control, cellular stress responses, intra- and intercellular communication and metabolic processes (Schimmel, 2018; Tian et al., 2022; Wilson and Dutta, 2022; Zhao et al., 2025).

### 2.1 Gene expression regulation

tRNA-derived fragments can regulate gene expression through RNA interference (RNAi)-like mechanisms. Several tRFs have been shown to target mRNAs via sequence complementarity, recruiting Argonaute (AGO) proteins and leading to transcript degradation or translational repression (Kuscu et al., 2018; Lyons et al., 2018; Tian et al., 2022). In addition, many tRFs were discovered to inversely modulate essential genes associated with AD and PD through a microRNA (miRNA)-like mechanism, in the SAMP8 mouse model of cerebral aging. Significantly, downregulated tRFs such as AS-tDR-011389 and AS-tDR-013428 were associated with the upregulation of pathogenic targets including Camk2n1, P2ry1, and Rpsa, whereas upregulated tRFs like AS-tDR-011775 targeted genes related to neurodegeneration, such as Park2 and Mobp, indicating their active role in synaptic dysfunction, amyloid toxicity, and astroglial signaling (Zhang et al., 2019). By directly disrupting translation initiation via eIF4F displacement and suppressing nascent protein synthesis through Ago2-mediated RNA silencing, certain tRF species, especially 5′tiRNAs and tRF-3s, contribute to neuronal necrosis. These tRFs exhibited cytotoxic effects in both primary neurons and models of ischemic stroke (IS), drawing attention to their function in stress-induced neuronal injury, ribosomal stalling, and translational repression. According to the results, neurodegenerative disorders associated with excitotoxicity and oxidative stress may have a new pathogenic mechanism and potential treatment target in aberrant RNA polymerase III-driven tRF expression (Cao et al., 2021). Besides, tRF-Ala-AGC-3-M8 reduces neuroinflammation and neuronal damage in AD by inhibiting the ERK1/2-p70S6K signaling cascade and targeting EphA7 (Deng et al., 2025). Neuroprotection and therapeutic possibilities in AD are offered by its restoration, which decreases glial activation and pro-inflammatory cytokine release. These post-transcriptional regulatory abilities position tRFs as potent modulators of neuronal gene expression in response to stressors commonly observed in neurodegenerative contexts.

### 2.2 Altering mRNA stability

Through binding to RNA-binding proteins or untranslated regions (UTRs) of mRNAs, tRFs can prevent degradation, leading to increased half-life and elevated protein expression of critical neuronal genes (Lyons et al., 2018; Zhao et al., 2025). This dualistic function underscores the complex regulatory roles of tRFs in the CNS. Supporting this, in the SAMP8 mouse model, several tRFs were identified as stabilizers of mRNAs associated with synaptic function and neuroprotection, indicating their significance in preserving neuronal integrity during aging and neurodegeneration (Kuscu et al., 2018; Zhang et al., 2019, 2023; Green et al., 2020). Moreover, translation control can be achieved via the Leu-CAG derived 3′ tRF improves the translation of mRNAs called RPS28 and RPS15, via binding of the tRF which in turn promotes the maturation of 18S ribosomal RNA (rRNA) and the assembly of 40S ribosomal subunits (Kim et al., 2017).

### 2.3 Translation regulation

Under cellular stress, tRFs, especially those derived from tRNA-Gly and tRNA-Val, can directly block translation initiation without requiring AGO association. This mechanism likely contributes to the adaptive translational reprogramming observed in neurodegeneration (Lyons et al., 2018). Beyond initiation blockade, tRFs exert fine-tuned control over ribosome activity. For example, some 3′ tRFs like tRF3003a, associate with polysomes to modulate elongation or termination rates of translation, potentially affecting synaptic plasticity and protein aggregation pathways relevant to AD and PD (Tian et al., 2022; Kim et al., 2025; Oberbauer et al., 2025; Zhao et al., 2025). Also, tiRNAs have been shown to displace eIF4G/A from mRNA caps, forming G-quadruplex (G4) structures that inhibit the assembly of the eIF4F translation initiation complex. This suppresses cap-dependent translation, a conserved response in ND models under oxidative stress (Ivanov et al., 2011; Li D. et al., 2024). Another study found that the formation of G4 structures by ANG-induced 5′ tiRNAs, especially from tRNA-Ala and tRNA-Cys, displaces eIF4F from mRNAs and inhibits translation initiation (Ivanov et al., 2014). By influencing ribosome biogenesis or translation factors, tRFs can modulate the capacity and fidelity of protein synthesis in neurons, affecting synaptic strength, memory consolidation, and cellular resilience (Lyons et al., 2018).

#### 2.3.1 Binding to argonaute

tRNA-derived fragments, especially 5′ tRFs and 3′ tRFs, can load onto Argonaute proteins (AGO1–4), functioning similarly to miRNAs in silencing gene expression through sequence-specific targeting. The tRF-AGO complex can inhibit target mRNA by cleavage or translational repression, a mechanism that is increasingly appreciated in neuronal and glial cells (Maute et al., 2013; Kumar et al., 2014). These fragments can associate with AGO complexes and inhibit mRNA expression via seed-sequence complementarity, indicating a Dicer-independent mechanism of post-transcriptional gene silencing that may be utilized in neurodegenerative disorders and stress-induced inflammatory conditions (Lyons et al., 2018; Green et al., 2020; Li D. et al., 2024).

#### 2.3.2 Acting on ribosomes

Certain tRFs can directly associate with ribosomal subunits, altering translation dynamics independent of mRNA silencing. For example, tiRNAs bind to 40S subunits and inhibit global protein synthesis, allowing selective translation of stress-responsive genes (Lyons et al., 2018). Additionally, tRFs can interact with the initiation factor eIF4F to inhibit cap dependent protein translation and ribosome assembly which in turn leads to impaired recovery of cells during stress conditions (Prehn and Jirström, 2020). Moreover, 3′ tRF-Leu-CAG remodels the secondary structure of ribosomal protein S28 (RPS28) and accelerates its synthesis, promotes ribosome biogenesis, and sustains translational capacity under proteostatic stress (Kim et al., 2017; Kim H. K. et al., 2019).

### 2.4 Epigenetic regulation and environmental factors

Emerging research shows that tRF expression profiles can be altered by aging, physical activity, and dietary habits. High-fat diets, for instance, disrupt normal tRF signaling pathways, leading to metabolic and inflammatory dysfunction in the brain (Lyons et al., 2018). A mice study revealed that sperm-derived 5′ tsRNA-Gly-GCC is upregulated in response to a paternal high-fat diet, acting as an epigenetic carrier that enhances gluconeogenesis in offspring by downregulating hepatic Sirt6 through direct interaction with its 3′ UTR, thus activating the FoxO1 pathway and facilitating intergenerational transmission of metabolic dysfunction and impairment of glucose metabolisms in the offsprings via epigenetic alterations (Wei et al., 2014; Wang et al., 2022). Expression patterns of tRFs change among monozygotic twins, indicating epigenetic and regulatory variations distinctive to each individual, even though they share the same DNA. When traditional genetic methods fail, these tRF signals can step in as forensic biomarkers to reliably differentiate between identical twins (Tian et al., 2025). Fertilization occurs when certain tRFs such 5′ tRF-Gly-GCC and 5′ tRF-Glu-CTC are produced and loaded into sperm. Intergenerational epigenetic inheritance and metabolic reprogramming in children are influenced by changes in the number of tRFs caused by a paternal low-protein diet. These tRFs regulate gene expression in the early embryonic stage, especially in metabolic pathways (Sharma et al., 2016). Additional publication proved that ANG-mediated production of tRF-5, 5′ tRF-Gly, 5′ tRF-Glu, and 5′ tRF-Val accumulates paternal inflammation and causes metabolic disorders by altering gene expression networks involved in glucose homeostasis and adiposity (increased fat/muscle ratio) in next generations (Zhang Y. et al., 2021).

tRNA-derived fragments also regulate the epigenome by influencing chromatin remodeling, DNA methylation, and histone modification. They interact with chromatin regulators or affect the transcription of epigenetic enzymes, thereby modulating long-term gene expression patterns relevant to synaptic plasticity and memory (Lyons et al., 2018; Cai et al., 2024; Tahiri et al., 2025; Zhao et al., 2025). Environmental factors such as hormone therapy and antidepressant use have been shown to modulate tRF expression in the brain (Martens-Uzunova et al., 2021). These treatments influence stress resilience and cognition by altering tRF-mediated epigenetic and translational programs (Wang et al., 2025).

### 2.5 Role of tRF in stress granules

Stress granules (SGs) are non-membranous cytoplasmic aggregates composed of proteins and RNAs that form in response to various cellular stress conditions (Marcelo et al., 2021). In response to environmental stressors, mammalian cells initiate a transcriptional and translational reprogramming characterized by the downregulation of housekeeping gene expression and the upregulation of genes involved in stress adaptation (Rawat et al., 2021). This adaptive response is initiated by translational inhibition mediated through the phosphorylation of eukaryotic initiation factor 2 alpha (eIF2α) and is further facilitated by the active sequestration of untranslated mRNAs into SGs. Both natural and synthetic 5′ tRFs induce SGs via an eIF2α-independent mechanism which highlights the effect of 5′ tRFs on translational repression pathways (Emara et al., 2010). Another study proved that G4-tiRNAs can regulate protein functions by mediating neuroprotection by recruiting YBX1 to aid in the construction of SGs, which in turn promote cell survival in stressful conditions by protecting neurons in the hippocampus of early onset AD (EOAD) patients and slow down the atrophy (Ivanov et al., 2014; Wu et al., 2021). Stress granule formation via ANG-induced 5′ tRFs can also proves the effect of these fragments on NLRP3 inflammasome activation regulation (Cai et al., 2024).

### 2.6 Receptor-mediated and ligand-like functions

#### 2.6.1 Toll-like receptor activation

Small non-coding RNAs (sncRNAs), including tRFs, have been reported to activate pattern recognition receptors such as Toll-like receptors (TLR7 and TLR8) (Pawar et al., 2024). For example, tDR-1:34-His-GTG-1 (5′ tRNA-His-GUG half) promotes Tumor Necrosis Factor-α (TNF-α) and Interleukin-1β (IL-1β) secretion via TLR7 activation. Such activation can induce neuroinflammatory cascades by stimulating microglia or peripheral immune cells, contributing to chronic inflammation in AD and ALS models (Yu et al., 2025). For example, tRFs are able to bind to TLRs directly to trigger inflammatory reactions from T-cells (Wang et al., 2006).

#### 2.6.2 Ligand activity

Certain extracellular tRFs act as ligands for surface or intracellular receptors, potentially modulating intercellular communication within the CNS. Although direct ligand-receptor interactions remain to be fully elucidated, tRFs secreted via extracellular vesicles (EVs) may mimic signaling molecules and regulate cellular responses (Torres and Martí, 2021; Weng et al., 2022; Cooke et al., 2024). Besides, tRFs bind to transmembrane proteins which has an effect on early neural development (Yuan et al., 2021).

### 2.7 Regulation of cellular responses

#### 2.7.1 Apoptosis

Several studies have demonstrated that specific tRFs can suppress pro-apoptotic genes or enhance anti-apoptotic pathways via gene silencing or protein modulation. This cytoprotective effect has been observed in models of ischemia, PD, and excitotoxicity (Lyons et al., 2018; Lv et al., 2024). tRFs can directly bind to cytochrome C (cyt C) either to form cyt C ribonucleoprotein complex or to inhibit caspase-9 activation in order to inhibit apoptosome formation (Su et al., 2020b). Another function of these fragments can be achieved via the regulation of other related gene expressions relate to apoptosis associated protein transport (Li D. et al., 2024). Moreover, tRFs can regulate neuroprotective factor expression and inhibit cell death pathways in conditions like brain tissue damage for the purpose of tissue recovery (Yuan et al., 2024).

#### 2.7.2 Inflammation

In neuroinflammatory models, tRFs have been implicated in the regulation of cytokine expression and microglial activation. Some tRFs suppress Nuclear Factor kappa B (NF-κB) signaling, while others enhance IL-6 or TNFα production depending on the context (Qiu et al., 2024). Several tRFs have been shown to regulate inflammation by modulating cytokine production and inflammatory signaling pathways. One of which is tDR-59:75-Thr-AGT-1-M2 (3′ tRF-Thr-AGT) that attaches to 3′ UTR of Z-DNA-binding protein 1 (ZBP1) which activates NLRP3 inflammasome to induce pyroptotic cell death (Sun et al., 2021). Another examples are IL-6 cytokine suppression by JAK3 inhibition via tDR-60:76-Cys-GCA-2-M7 and IL-8 enhancement by NF-κB p65 activation via tDR-1:34-Ala-CGC-1-M3-D22GC-A25G-U26C (5′ tRF-Ala-CGC) (Liu et al., 2018; Green et al., 2020).

#### 2.7.3 Role of mitochondrial tRFs on mitochondria function

Mitochondrial tRFs (mt-tRFs) are increasingly recognized as important regulators of cellular metabolism. In neurodegenerative contexts, mt-tRFs are generally downregulated in AD mice, contributing to mitochondrial dysfunction which is a hallmark of diseases like PD and AD (Zhang et al., 2024). These fragments may modulate oxidative phosphorylation (OXPHOS) or mitochondrial translation by targeting mitochondrial mRNAs or ribosomal proteins. For instance, the mitochondrial tRFs, mt-tRF-Leu-TAA, promotes the translation of electron transport chain components, which in turn boosts OXPHOS. The synthesis of ATP is enhanced and the release of insulin in pancreatic β-cells is coupled with mitochondrial metabolism in response to glucose. Thus, mt-tRF-Leu-TAA can be a potential biomarker in NDs (Jacovetti et al., 2024). mt-tRFs can also mediate paternal epigenetic inheritance by transferring environmentally induced mitochondrial signals from sperm to the embryo, thereby altering early embryonic gene expression and influencing offspring metabolic phenotypes such as impaired glucose tolerance and insulin sensitivity in the offspring (Tomar et al., 2024). In addition to mitochondria-derived tRFs, nuclear-derived tRFs also participate in the regulation of mitochondrial functions. 5′ tsRNA-Glu-CTC, translocates from cytosol to mitochondria, reduces mt-tRNA-Leu association and impairing its aminoacylation by competitively binding to LaRs2, which in turn disrupting mitochondrial protein synthesis and function leading to memory decline (Li D. et al., 2024). In the cytoplasm, nuclear gene expression is modulated by mt-tRFs via interacting with RNA-binding proteins such as AGO, thereby regulating mRNA stability and translation. These interactions enable mt-tRFs to influence diverse cellular processes including metabolism, stress responses, and inter-organelle communication (Muneretto et al., 2024). For that matter, the ratio of nuclear-tRF/mt-tRF in cytoplasm can be a potential biomarker for the progresses of ND (Paldor et al., 2023; Madrer et al., 2025).

#### 2.7.4 Oxidative stress

Oxidative stress is a central driver of pathogenesis in neurodegenerative disorders, triggering extensive tRNA fragmentation as a cellular response (Deng et al., 2022). Stress factors such as heavy metal or H_2_O_2_ exposure, angiogenin-mediated cleavage of mature tRNAs increases the production of tRFs (Huang and Hopper, 2016; Chen and Liu, 2017; Hou et al., 2022). tRFs regulation is influenced in order to either protect the cell or pave the way for cell death. For example, tRF-Glu-CTC levels were regulated in both *in vivo* and *in vitro* experiments in response to H_2_O_2_ which has an effect on neurogenesis (Karacicek et al., 2025). On the other hand, tRFs can trigger oxidative stress. A 5′ tRF, tRF-Gly-GCC, is found to promote ROS production in radiation induced lung injury (Deng et al., 2022). As a result, oxidative stress not only escalates tRNA cleavage and dynamically modulates tRF levels some of which are upregulated to mediate stress adaptation, while others are downregulated to rebalance translation which is revealing a sophisticated mechanisms to maintain cellular homeostasis but also can be triggered by the tRFs which leads to disease progression.

## 3 Detection, prediction and verification tools

Scientists are now able to identify, categorize, and thoroughly study tRFs because of the fast advancement of small non-coding RNA sequencing (sncRNA-seq) technology. The field of tRF research is dependent on certain databases, identification tools, and verification procedures (Table 1).

**TABLE 1:** Bioinformatics, predictive, experimental and functional tools for tRNA derived fragment (tRF) research.

Method	Key strength	Key limitation
**Bioinformatic tools**		
MINTbase 2.0	Offers curated tRF sequence, abundance, and metadata across tissues and cancers	Only includes tRFs ≥ 16 nt. and for homo sapiens
tDRnamer/tRAX	Standardized naming system/High-confidence tRF quantification	Varies by algorithm; susceptible to tRNA misannotation. Only available for known tRFs
**Predictive tools**		
tRFtarget 2.0	Predicts for mRNA targets of tRFs using hybridization thermodynamics and CLASH data	Predictions need experimental validation
IntaRNA/RNA22	Predict interactions based on seed, accessibility, and free energy	Often used in combination for better specificity. miRNA bias, potential false positives, no tRF-specific tuning
**Experimental tools**		
Northern blotting	Accurate size confirmation and visual validation. Gold standard	Labor intensive and time consuming. Low sensitivity for low abundance tRFs
qRT-PCR	Sensitive and quantitative; condition specific. Faster and cheaper than sequencing	Requires primer optimization due to tRF short length & modifications. Only available for known tRFs
**Functional tools**		
Antisense inhibition	Specific knockdown of tRFs: reveals biological role	Off-target effects: delivery optimization needed
Luciferase assay	Validates direct gene targeting of tRFs via 3′ UTR binding. Sensitive.	Depends on predicted target sites
CLIP/RIP assays	Reveals *in vivo* RNA–protein interactions. Identification of RNA binding sites	Technically demanding, requires high-quality antibodies and UV crosslinking. Low yield, high background
RNA pulldown	Confirms tRF-target or tRF-protein interactions *in vitro*. Validation for mechanism of actions.	Requires biotin labeling, prone to non-specific binding

### 3.1 Identification tools

#### 3.1.1 Small RNA sequencing

Small non-coding RNA sequencing (sncRNA-seq) data including the steps of RNA isolation, bioinformatics analysis, library preparation, and sequencing, is the fundamental technique to identify tRFs. Common techniques for efficient RNA isolation include TRIzol or column-based procedures which are prerequisite for high-quality sncRNA-seq data. An essential part of capturing tRFs is selecting and enriching tiny tRNA fractions (14–50 nucleotides) according to their size. Library preparation must be completed, which includes adapter ligation, reverse transcription, and amplification to complete thorough high-throughput sequencing on Illumina systems to produce millions of short reads (Lee et al., 2009; Aalto and Pasquinelli, 2012). Specialized kits like the Arraystar rtStarTM tRF&tiRNA Pretreatment and First-Strand cDNA Synthesis Kit have been created to improve the accuracy of tRF identification by ensuring efficient removal of internal tRNA modifications and proceeding of reverse transcriptase during cDNA synthesis (Xu et al., 2024; Yu et al., 2024).

#### 3.1.2 Differential expression and functional annotation

Post-sequencing analysis includes cutting adapters, and mapping reads to reference genomes by using bioinformatics tools like Bowtie 2.0, STAR, or HISAT2 (Langmead and Salzberg, 2012; Dobin et al., 2013; Kim D. et al., 2019). To achieve tRF detection specificity, it is necessary to filter out contaminants like rRNA or miRNAs and find preset sequence patterns. Differential expression analyses, such DESeq2, amplify variations in tRF expression across trial conditions (Love et al., 2014). After examining differentially expressed tRFs, pathway and functional enrichment analyses such as Kyoto Encyclopedia of Genes and Genomes (KEGG) and Gene Ontology (GO) give further biological meaning.

tRNA-derived fragment research requires systematic categorization, nomenclature, and functional prediction capabilities provided by databases. MINTbase 2.0 stands out for its extensive database of all known tRFs for *Homo sapiens* (Pliatsika et al., 2018). This database allows researchers to easily explore, compare, and evaluate tRFs through its user-friendly interface among the other tRF cataloging sites. tRF identification may be performed in databases like tsRNAsearch for other species (Donovan et al., 2021). tDRnamer and the tRAX are the two advanced tools systematic tRF annotation and categorization (Chan et al., 2025). tRAX allows for the high-throughput processing and analysis of differential expression from small RNA sequencing data in order to efficiently identify tRFs for various species. On the other hand, tDRnamer uses machine learning methods to categorize tRFs according to sequence features and genetic origin. tDRnamer aimed to generate a consensus by providing consistent nomenclature and maintaining consistency between investigations. Therefore, scientists can use this tool to convert the tRF names by using the sequences of their selected tRFs.

#### 3.1.3 Target prediction tools

By integrating thermodynamic modeling with experimental data from crosslinking, ligation and sequencing of hybrids (CLASH) which is used to detect RNA-RNA interactions (Xie et al., 2020), tRFtarget 2.0 stands out as a web-based tool especially designed for predicting tRF-mRNA interactions, which is important for target prediction for tRFs (Li N. et al., 2024). In addition, tRFtarget performs pathway-level understanding by including downstream functional enrichment as well. On the other hand, there are two other methods that were initially made for miRNA research, RNA22 and IntaRNA, are tools but have now been modified to anticipate tRF targets (Miranda et al., 2006; Mann et al., 2017; Xie et al., 2020). This is because of their accessibility modeling and flexible seed-pairing procedures. With its focus on accessibility and interaction energy, IntaRNA provides strong predictions for both short and structured RNAs, whereas RNA22 excels at finding non-canonical interactions free of conservation bias. RNA22 were utilized for various studies; to investigate tRF targets for thyroid cancer study, for the validation of AGO-loaded tRFs by combining Cross-linking Immunopreciptation (CLIP) data, to find functional relationships of tsRNA-mRNA interactions in neurological damage (Kuscu et al., 2018; Li et al., 2020; Do et al., 2024). These methods have been utilized in a number of research. These techniques are used to make predictions regarding the regulatory potential of tRFs in many biological systems. They are trusted by many to help them test theories. When it comes to detecting non-canonical and seed-independent interactions, RNA22 provides more flexibility than tRFtarget 2.0, which is optimized for tRF-specific interactions using experimentally informed parameters. On the other hand, IntaRNA is great for structured RNA targets because of its emphasis on accessibility and energy-based modeling. As a result, it can be said that they complement each other well; tRFtarget 2.0 is very specific, RNA22 is very exploratory, and IntaRNA is very structurally precise.

#### 3.1.4 Quantitative reverse transcription PCR (qRT-pCR)

Quick, sensitive, and precise detection of tRFs is possible with PCR-based techniques like quantitative reverse transcription PCR (qRT-PCR) (Boskovic et al., 2020; Xie et al., 2020). The rapidity, low cost, and high sensitivity of this experimental method make it an attractive approach for quantifying tRFs with low abundance. On the other hand, PCR relies on pre-existing sequence information, which can lead to amplification bias, and it might be challenging to discover new or unexpected tRF sequences. Thus, due to their tiny size and structural variability, tRFs need careful primer design for qRT-PCR validation in order to distinguish between related isoforms and guarantee accurate quantification to find a mechanistic link was found between altered tRNA processing and tRF accumulation in neurological disorders (Wu et al., 2021; Yu et al., 2024).

#### 3.1.5 Northern blot

Northern blotting provides visual confirmation that supplements sequencing-based predictions by validating the existence, length, and expression pattern of tRFs (Xie et al., 2020; Su et al., 2022; Esmaeili et al., 2025). For early-stage discovery and stress/disease-specific tRF response analysis, it is still the gold-standard approach for differentiating physiologically produced tRFs from degradation artifacts (Boskovic et al., 2020; Wu et al., 2021).

### 3.2 Verification

#### 3.2.1 Antisense inhibition and AAV-shRNAs

Researchers often employ antisense oligonucleotide-based inhibition to validate tRFs and understand the biological effects of species depletion. Locked nucleic acid (LNA) modified antisense oligonucleotides are highly effective at targeting sRNA molecules, such as tRFs, because of their high binding affinity and nuclease resistance (Su et al., 2022). One way to study cell survival, stress responses, apoptosis, and global translation rates after tRF knockdown is by incorporating these inhibitors into cultured cells or model organisms. Then, one can use Western blotting, flow cytometry, and ribopuromycylation to measure these outcomes (Mahmood and Yang, 2012; Bastide et al., 2018; McKinnon, 2018). Transcriptome profiling and antisense inhibition demonstrate the significance of tRF-regulated downstream gene networks in disease-relevant biological processes like neuroinflammation, metabolic regulation, and tumor development, and the fact that their inhibition enhances apoptosis in neurons subjected to oxidative stress suggests that certain 5′ tiRNAs produced from-(ANG) play a protective role in cellular homeostasis.

Adeno-associated Virus mediated short hairpin RNA (AAV-shRNA) knockdown method employs a construct to inhibit tRF expression in cellular or animal models. AAV-shRNA is critical step for experimental validation of computational predictions and assurance of their biological relevance (Monahan et al., 2002; Moore et al., 2010). AAV-shRNA knockdown may evaluate phenotypic consequences by analyzing cellular activity, changes in gene expression, and downstream pathways, which may provide light on the functional roles of tRFs.

#### 3.2.2 Luciferase reporter assays

Another functional verification method, luciferase reporter assay, examines the interactions between tRFs and mRNAs to provide evidences of gene expression regulations by measuring how tRF binding to the 3′ UTR of target mRNAs modulates luciferase activity (Tong et al., 2020; Su et al., 2022; Esmaeili et al., 2025). As an example, firefly luciferase activity was significantly reduced by tRF-Ala-AGC-3-M8 mimic compared to the negative control when using the wild-type reporter, stating that this tRF directly represses gene expression via interaction with the reporter’s target sequence (Deng et al., 2025).

#### 3.2.3 CLIP/RIP

Crosslinking and immunoprecipitation (CLIP) and its variant RNA immunoprecipitation (RIP) are powerful approaches to experimentally validate the interactions between tRFs and RNA-binding proteins (RBPs), particularly those involved in post-transcriptional regulation. Photoactivatable-Ribonucleoside-Enhanced CLIP (PAR-CLIP) is another powerful technique that combines metabolic labeling with UV crosslinking to capture RNA-protein interactions with high specificity and single nucleotide resolution, whereas RIP and CLIP have lower resolution or efficiency but are simpler to perform (Xie et al., 2020). These tests identify the presence or absence of certain tRFs in various biological components, such as effector complexes including AGO proteins or RBPs containing YBX1, which plays a role in stress granule formation (Lyons et al., 2016). For example, interaction of tRF-3b with AGO proteins was confirmed by RIP, supporting the idea that it regulates gene expression through a miRNA-like, AGO-dependent mechanism (Su et al., 2022). The bound sRNA species are identified by target RBP immunoprecipitation, RNA extraction, and high-throughput sequencing such as CLIP-seq after UV crosslinking covalently stabilizes RNA-protein interactions *in vivo*. In order to identify more stable RNA-protein interactions in their natural environment, RIP employs a comparable method that does not include UV crosslinking. The physical binding of tRFs to regulatory proteins and their functional pathways are confirmed by these methodologies. Findings from CLIP-based studies point to the roles of certain tRF-5 in mRNA targeting and translational control, since these proteins are attracted to AGO2 complexes in neurological diseases.

#### 3.2.4 RNA pulldown assay

For the purpose of validating anticipated tRF interactions with target proteins or RNAs, RNA pulldown experiments are potent experimental methods. This technique involves capturing interacting molecules by incubating synthetic tRF probes tagged with biotin with cellular lysates. The complexes are separated by using magnetic beads coated with streptavidin, which are subsequently subjected to a battery of washing and elution procedures. Mass spectrometry and next-generation sequencing are used to identify the proteins and RNAs that have been captured, respectively. RNA pull-down assays were used to detect tRF interactions with spliceosomal components contributing to neurodegenerative phenotypes, ribosomal complexes regulating translation under neurodegenerative stress in human samples, hnRNPF proteins modulating RNA splicing and histone gene regulation, stress granule proteins such as YB-1, TIA1, and G3BP1, promoting stress granule assembly and contributing to neuronal survival mechanisms during neurodegenerative stress (Dubnov et al., 2024; Madrer et al., 2025; Boskovic et al., 2020; Prehn and Jirström, 2020). This method clearly establishes direct contacts, shedding light on the regulatory networks and biological processes mediated by certain tRFs.

## 4 The role of tRNA modifying enzymes in neurological disorders

The impairments in tRNA metabolism are associated with brain abnormalities and neurological disorders (Qin et al., 2020). Mutations in certain genes that encode enzymes such as ANG, cleavage factor polyribonucleotide kinase subunit 1 (CLP1) and NOL1/NOP2/SUN domain tRNA cytosine methyltransferase (Nsun2) have been implicated in disruptions of tRNA biogenesis.

Angiogenin in its mature form contains three key structural elements that determine its function: a receptor-binding site, a catalytic region with ribonuclease activity, and a signal sequence for nuclear entry. These three components are required for ANG to work efficiently. Disruptions to ANG’s structure may impair both its RNA-cleaving function and nuclear import (Tripolszki et al., 2019). Mutations in the ANG gene that alter its activity have been shown to be linked to both sporadic and familial ALS cases (Greenway et al., 2006). This finding was followed by the identification of ANG mutations in ALS patients from various regions worldwide (Aparicio-Erriu and Prehn, 2012). The literature on ANG mutations in ALS has been expanding considerably in recent years, and ongoing studies continue to enhance our understanding of ANG’s involvement in this field (Prehn and Jirström, 2020). ANG has been shown to play a neuroprotective role under stress conditions both *in vivo* and *in vitro* (Kieran et al., 2008; Skorupa et al., 2012; Hogg et al., 2020). Specifically, ANG provides neuroprotective effects against hypoxic injury of motor neurons. However, this neuroprotective phenomenon is lost in ALS-associated ANG mutations including K40I, Q12L, K17I, R31K, C39W, and I46V as demonstrated in NSC34 cells (Sebastià et al., 2009). In a separate study, this protection is suggested to occur through ANG’s cleavage of tRNA to generate tiRNAs. Hogg et al. (2020) suggested that the serum 5′ tRF-Val-CAC might hold biomarker potential in ALS and be related to activation of neuroprotection under stress. In their study, MZ-294 cells were treated with ANG, and small RNA sequencing followed by custom bioinformatics analysis was performed to characterize tRFs produced by ANG. A significantly different profile was observed between ANG-treated and vehicle control groups for the 5′ tRF-Val-CAC (Hogg et al., 2020). Besides its neuroprotective role, ANG has been shown to play a role in neurite pathfinding, as demonstrated when the ribonucleolytic activity of human ANG was inhibited by NCI 65828 inhibitor in P19-derived neurons. In 2019, a research group examined the ANG gene through sequencing by comparing 136 sporadic ALS patients to 112 healthy controls of Hungarian origin. Among the mutations found in the analysis, R33W mutation led to partial reduction in both ribonucleolytic activity and nuclear translocation efficiency of ANG (Tripolszki et al., 2019). ANG mutations were also detected in patients with other NDs such as AD and PD. For example, 1,001 AD patients and 1,010 control cohort with Italian origins were investigated for ANG gene mutations. The results revealed a non-sense mutation (K73X) which made the mature protein 51 amino acids shorter and diminished the catalytic functional site in two AD patients (Gagliardi et al., 2019). Additionally, ANG mutations including K17I and I46V have been identified in PD patients from different ethnic backgrounds. The specific ANG variants detected appear to vary according to ethnic origin (van Es et al., 2011; Prehn and Jirström, 2020).

Methyltransferase is a key factor in post-transcriptional modifications of RNAs by introducing 5-methylcytosine (m5C) (Van Haute et al., 2019). Mutations in NSun2 lead to intellectual disability and facial dysmorphism, highlighting its critical role in human neurodevelopmental processes (Abbasi-Moheb et al., 2012; Khan et al., 2012). These pathological findings were associated with Dubowitz Syndrome characterized by microcephaly and intellectual disability (Martinez et al., 2012). In NSun2-deficient mice and patient fibroblasts, loss of NSun2 resulted in the ANG-mediated cleavage of specific tRNA isotypes (Asp, Glu, Gly, His, Lys, Val) and accumulation of tRF-5 (Blanco et al., 2014). Increased tRF-5 leads to disruption in protein translation and induction of stress pathways. Moreover, neuronal death was observed in cortical, hippocampal, and striatal neurons. The inhibition of ANG was sufficient to rescue the phenotype of NSun2-deficient mice. Together, these findings demonstrate that impaired tRNA methylation caused by NSun2 deficiency can lead to abnormal activation of ANG, resulting in the accumulation of tRF-5 and ultimately disrupting neuronal development and survival. Another study reported that NSun2 activity is essential for neuronal differentiation (Flores et al., 2017). Loss of function in NSun2 resulted in the accumulation of intermediate progenitor cells and a reduction in differentiated upper-layer cortical neurons. Together with reduced NSun2 expression, exposure to recombinant (ANG) exacerbates the impairment of neuronal differentiation. The NSun2 deficiency is also associated with synaptic transmission and depression (Blaze et al., 2021). The deficiency of NSun2 specifically in the forebrain resulted in reduced Gly tRNA levels and downregulation of synaptic protein translation. Taken together, NSun2 plays a pivotal role in tRNA modifications, neuronal differentiation and synaptic plasticity whereas its loss contributes to pathophysiology of neurodevelopmental disorders.

Cleavage factor polyribonucleotide kinase subunit 1 is RNA kinase unique to mammals and has a role in tRNA splicing through interaction with the tRNA splicing endonuclease (TSEN) complex (Hanada et al., 2013). The loss of kinase activity of CLP1 leads to death of spinal motor neurons, deficits in motor control and paralysis. Furthermore, dysregulation of CLP1 results in an increase in tyrosine (Tyr) tRFs *in vitro*. The accumulation of Tyr-tRFs was also present in the spinal cord and cortex of CLP1 kinase-dead mice. Supporting this finding, another study demonstrated that the mature tRNAs were depleted while intron-containing the amount of pre-tRNAs was increased in induced neurons generated from CLP1 mutant patients (Schaffer et al., 2014). The transfection of unphosphorylated 3′-tiRNAs rendered CLP1 mutant neurons susceptible to oxidative stress-induced cell death (Schaffer et al., 2014). In another study, oxidative stress increases the production of Tyr-tRFs in both wild-type and CLP1 mutant mouse embryonic fibroblasts (MEFs) (Hanada et al., 2013). The defect in CLP1 increased the loss of motor neurons. The accumulation of 5′ exon of tyrosine pre-tRFs (5′ Tyr-tRF) negatively regulates neural differentiation by enhancing the cell death in SH-SY5Y human neuroblastoma cell line (Inoue et al., 2020). The injection of 5′ Tyr-tRF resulted in microcephaly in zebrafish larvae and reduced number of spinal motor neurons in the spinal cord. The p53 inactivation rescued the phenotype associated with the CLP1 mutation *in vitro and in vivo* (Hanada et al., 2013; Inoue et al., 2020). These findings indicate that the oxidative stress-induced cell death is through a p53-dependent pathway. PKM2 (Pyruvate Kinase M2) has been demonstrated as a target of 5′Tyr-tRF *in vitro* and in zebrafish (Inoue et al., 2020). These results indicate that the interaction between 5′ Tyr-tRF and PKM2 during neurogenesis may serve as an initiating factor for neuronal dysfunction. Additionally, a recent study has identified PKM2 as a critical factor involved in the regulation of neurogenesis (He et al., 2025).

Human CLP1 mutation (R140H) was detected in five genetically distinct families (Karaca et al., 2014). The CLP1 (R140H) mutation does not abolish the kinase activity of CLP1. However, it specifically disrupts the interaction with the TSEN complex. Similar to the findings, another group found that nuclear localization and kinase activity of CLP1 were reduced (Schaffer et al., 2014). The patients carrying the CLP1 mutation and CLP1 kinase-dead mice exhibit microcephaly and deficits in motor and sensory function (Karaca et al., 2014). In another study, patients from four unrelated families demonstrate clinical features similar to Pontocerebellar hypoplasia (PCH), a disease defined by atrophy of the cerebellum and brainstem (Schaffer et al., 2014). In conclusion, impaired CLP1 activity perturbs tRNA biogenesis, leading to neurodevelopmental disorders and NDs.

## 5 tsRNAs in neurological disorders

**FIGURE 2:**
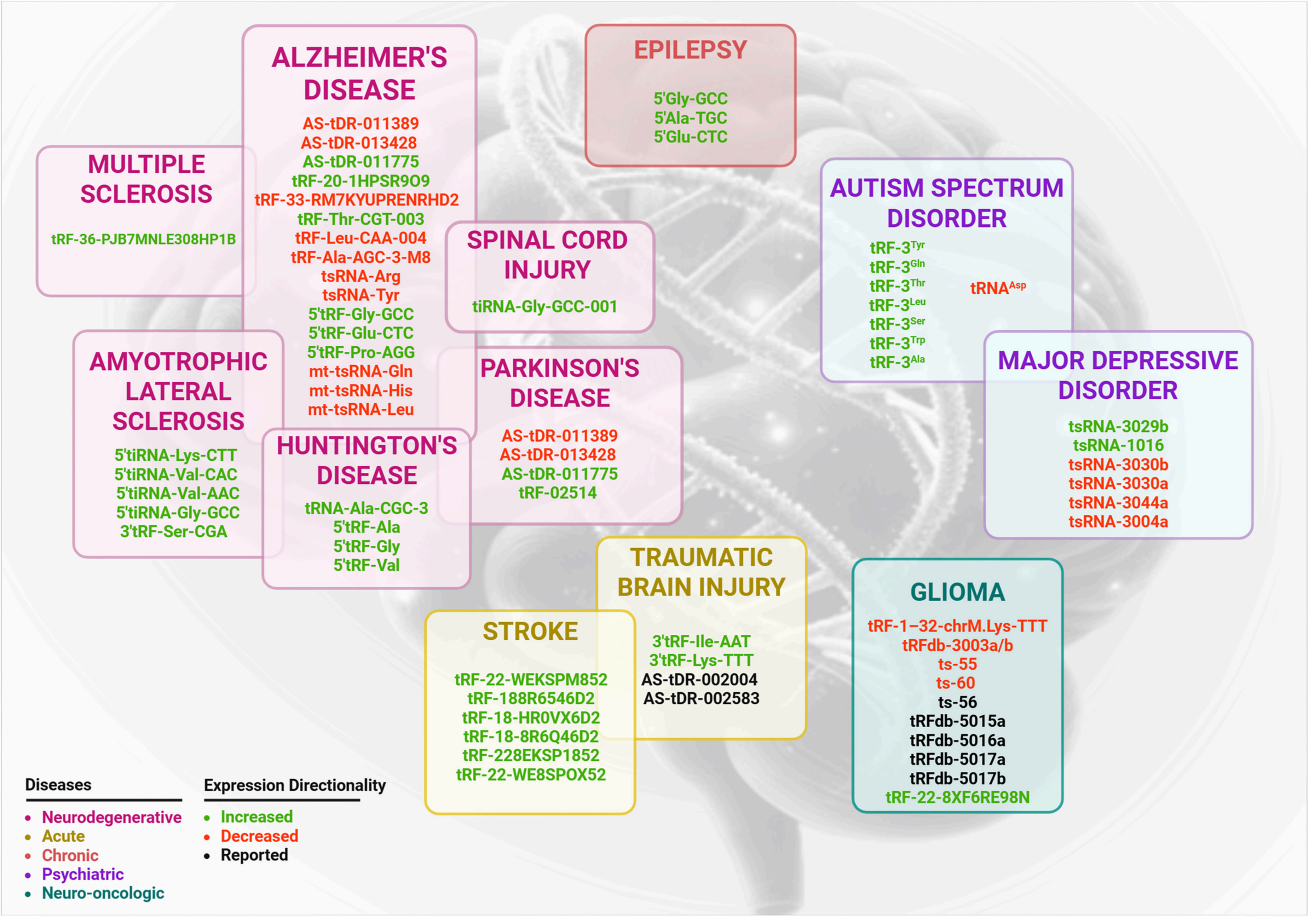
Differentially regulated tsRNA associated with neurological and neuropsychiatric disorders. This schematic summarizes expression of tsRNA reported in the literature and each disease category grouped in separate boxes according to pathological classifications: neurodegenerative (magenta), acute (yellow), chronic (orange), psychiatric (purple) and neuro-oncologic (teal). Expression directionality is color coded: green for increased expression, red for decreased expression and black for for tRFs with unclear or undetermined regulation direction.

Growing data from *in vivo* animal models, *in vitro* systems, and clinical studies in patients has showed that variations in tRF expression patterns are correlated with neuroinflammation and neuronal dysfunction (Supplementary Tables 1–3). tRF subtypes are considered both functional mediators in pathogenesis and valuable promising biomarkers for neurological disorders (Figure 2). Therefore, understanding the diverse role of specific tRF subtypes across neurological conditions is essential for developing targeted therapeutic strategies and enhancing precision medicine approaches in this field.

### 5.1 Alzheimer’s disease

Alzheimer’s disease is a progressive neurodegenerative disorder characterized by extracellular amyloid-beta (Aβ) protein aggregation creating senile plaques and intracellular accumulation of hyperphosphorylated tau protein, which forms neurofibrillary tangles (Ashrafian et al., 2021). Accumulating evidence indicates that sncRNAs such as tRFs have emerged as novel diagnostic tools, therapeutic targets and regulatory molecules in the pathophysiology of AD.

Several studies investigating molecular basis and pathogenesis of AD revealed changes in tRF levels across various brain regions and biological samples. For example, in small neuron-derived extracellular vesicles (sNDEVs) derived from serum samples of AD patients, three tRFs were significantly upregulated while 10 tRFs were downregulated. Among them changes in tRF-20-1HPSR9O9 and tRF-33-RM7KYUPRENRHD2 expression confirmed by qRT-PCR suggest that these sNDEV-derived tRFs may represent promising biomarkers for distinguishing familial Alzheimer’s disease from sporadic cases and healthy controls. Also tRF-20-1HPSR9O9 expression in sNDEVs was inversely correlated with p-Tau levels in AD patients (*P* = 0.041, *R*^2^ = −0.3473) (Arioz et al., 2025). Similarly, AD patient brain tissues showed reduced levels of tsRNA-Tyr and tsRNA-Arg in the frontal lobe cortex and increased levels of 5′tRF-Gly-GCC, 5′ tRF-Glu-CTC, and 5′ tRF-Pro-AGG in the hippocampus. These dysregulated tsRNAs are associated with neurodevelopmental pathways such as synapse formation and have been implicated in increasing the susceptibility of motor neurons to oxidative stress–induced cell death, highlighting their involvement in both physiological motor neuron functions and stress-related responses (Zhang et al., 2020). Additionally, 5′ tRF-Pro-AGG correlated with disease stage progression, suggesting its potential as a diagnostic biomarker for monitoring AD onset and development (Wu et al., 2021, 2023).

Another study emphasized sex-specific differences, reporting significant reductions in tRFs targeting cholinergic transcripts (CholinotRFs) in the nucleus accumbens of female AD patients. Interestingly, five of these tRFs derived from the same mitochondria-derived tRNA, PheGAA (Shulman et al., 2023). Supporting this sex-specific pattern, Dubnov et al. (2024) showed that the level of tDR-36:75-Asn-GTT-2-M2 in the cerebrospinal fluid (CSF) of female AD patients was elevated compared to cognitively healthy controls, while the level of the same tRF showed no difference in male patients (Dubnov et al., 2024).

In animal models, altered tiRNA and tRF profiles were observed in the hippocampus of APP/PS1 mice (Lu et al., 2021). Differential expression levels of tRF-Thr-CGT-003 and tRF-Leu-CAA-004 validated by qRT-PCR and they were identified as regulators of calcium-related proteins, including the voltage-gated calcium channel γ2 subunit and the endoplasmic reticulum calcium release protein RYR1, as well as retinol metabolism–associated enzymes such as CYP2S1 and CYP2C68. The use of RNA modification aborted sequencing (PANDORA-seq) revealed downregulation of mitochondrial tsRNAs in the prefrontal cortex of 5xFAD mice, with three of them (mt-tsRNA-Gln, mt-tsRNA-His, and mt-tsRNA-Leu) were significantly decreased (Zhang et al., 2025). Additionally, the traditional Chinese medicine Bushen Tiansui formula (BSTSF) modulated 57 tsRNAs in right hippocampus of AD rats. KEGG pathway and GO analyses find potential therapeutic mechanisms through which tsRNAs exert. Treatment-associated tsRNAs may mediate their beneficial effects by modulating the cAMP signaling pathway and retrograde endocannabinoid signaling pathways (Zhang Z.-Y. et al., 2021). Collectively, these studies highlight region- and sex-specific alterations in tRF expression in AD, but a universally consistent tRF biomarker across studies has not yet been established.

### 5.2 Parkinson’s disease

Parkinson’s disease is characterized by the progressive degeneration and loss of dopaminergic neurons in the *substantia nigra pars compacta*, as well as catecholaminergic neurons in the *locus coeruleus* (Prehn and Jirström, 2020). High-throughput small RNA sequencing of post-mortem brain samples from both premotor and motor stages of PD, along with age-matched control subjects, was used to quantify the various types of sRNAs in the amygdala. Among the most sequenced RNAs, tRNAs contributed significantly, accounting for 24% of the total, with tRNA-Val-GTY being the most highly expressed tRNA (Pantano et al., 2016). In a re-analysis of 254 RNA-seq datasets from three previous studies, researchers examined tRF expression in prefrontal cortex, CSF, and serum samples from male and female PD patients and controls. A total of 62 differentially expressed tRFs were found to be shared between CSF and the prefrontal cortex. Additionally, sex-dependent differences in tRF expression were observed between PD patients with and without dementia in both CSF and serum samples (Magee et al., 2019). Magee et al. (2019) also identified a subset of 5′-tiRNAs as smallest sets of tRFs that could serve as distinguishing biomarkers. These 5′-tiRNAs are already known to function in neuronal processes in ALS (Ivanov et al., 2011). They may play a protective role in PD, potentially influencing neuronal survival under stress conditions. Another study re-analyzed short RNA-seq data from post-mortem CSF and blood samples of PD patients and controls. The results revealed elevated levels of long tRFs in CSF compared to blood. CSF also showed a notable age-related decline in specific 3′- and internal tRFs, along with more pronounced sex-specific differences than those observed in blood (Paldor et al., 2023). Specifically, mitochondrial tRFs were enriched in sequences targeting cholinergic transcripts, suggesting that mitochondrial loss, a hallmark of PD, may reduce tRFs. Therefore, it might increase cholinergic transcript levels and disrupting cholinergic balance. Another study re-analyzed short RNA-seq data from post-mortem CSF and blood samples of PD patients and controls. The results revealed elevated levels of long tRFs in CSF compared to blood. CSF also showed a notable age-related decline in specific 3′- and internal tRFs, along with more pronounced sex-specific differences than those observed in blood (Paldor et al., 2023). Furthermore, the analysis of EVs derived from the serum of PD patients identified 122 upregulated and 69 downregulated tRFs, with tRF-02514 showing the highest expression level. Functional studies demonstrated that tRF-02514 promotes microglial pyroptosis and neuroinflammation by suppressing ATG5-mediated autophagy. Notably, knockdown of tRF-02514 in a PD mouse model reduced neuroinflammation, enhanced autophagy, and alleviated PD-related pathology (Dong et al., 2025). These findings point out tRF-02514 not only as a potential biomarker but also as a therapeutic target.

### 5.3 Amyotrophic lateral sclerosis

Amyotrophic lateral sclerosis is a terminal ND that is caused by the progressive degeneration and death of motor neurons in the CNS (Ye et al., 2025). While approximately 10% of ALS cases are linked to genetic factors, the majority of the cases occur sporadically without any clear family history (Rojas et al., 2020). Currently, clinical symptoms are used for diagnosis of the disease. However, the symptoms do not emerge until neurodegeneration is already underway (Witzel et al., 2022). Therefore, earlier detection of the disease and reducing treatment delays requires biomarker development.

Recent studies have highlighted the involvement of tRFs and tiRNAs in ALS progression and pathology. In a study, Hogg et al. (2020) measured spinal cord and serum samples obtained from *SOD1*^*G*93*A*^ mice with different genetic backgrounds in terms of disease progression and lifetime. The analysis revealed elevated 5′ Val-CAC concentrations in spinal cord tissue from slow-progressing *SOD1*^*G*93*A*^ mice compared to their fast-progressing counterparts. Additionally, they compared 5′ Val-CAC level from 16 slow- and 70 fast-progressing ALS patients along with 91 healthy controls. It was significantly elevated at symptom onset, correlating with angiogenin upregulation, altered translation initiation, and slower disease progression. Serum 5′ Val-CAC levels were elevated in ALS patients with slower disease progression, indicating its potential as a prognostic biomarker linked to angiogenin-mediated stress responses in motor neurons (Hogg et al., 2020). The same research team conducted small RNA sequencing in 2024 using spinal cord from SOD1^G93A^ and TDP43^A315T^ mouse models. The bioinformatics analysis demonstrated the upregulation of 5′ tiRNA-Lys-CTT in the SOD1 mouse model and 5′ tiRNA-Val-CAC and 5′ tiRNA-Val-AAC in the TDP43 mouse model. Researchers validated the significant increased level of 5′ tiRNA-Val-CAC in TDP43 mouse model by custom TaqMan assays. Since ANG generates these 5′ tiRNAs through cleavage at the anticodon loop, these results provide direct evidence that elevated tiRNA levels direct consequence of enhanced ANG activity in both mutant ALS mouse models. The accumulation of these tiRNAs highlights a mechanistic connection between ANG dysregulation, altered tRNA cleavage, and neuronal stress responses in ALS The small RNA sequencing results also showed decreased expression of seven 3′ tRFs in TDP43 mouse model compared to control while 3′ tRF-Ser-CGA was increased in the SOD1 mouse model. The authors proposed that the reduction in 3′ tRFs, which result from T loop cleavage of tRNA, may reflect impaired activity of Dicer or other RNases (Baindoor et al., 2024). In 2025, Jirström et al. (2025) reported a significant increase in 5′ tiRNA^Gly–GCC^ levels in the lumbar spinal cord of TDP-43^A315T^ mice. Although a similar elevation was observed in the SOD1^G93A^ and FUS (1–359) ALS mouse models, these increases were not statistically significant. Additionally, 5′tiRNA-Gly-GCC levels upregulated in primary cortical neurons exposed to ALS-related oxidative, proteasomal, and excitotoxic stress. They transfected the primary neurons with synthetic mimics of 5′ tiRNA-Gly-GCC and investigated its role by whole-transcript RNA sequencing and proteomics. The results revealed that majority of the mRNAs were downregulated and contained predicted 5′ tiRNA-Gly-GCC binding sites, pointing its role in silencing the target genes. Label-free mass spectrometry results showed downregulation of key proteins essential for translation initiation and ribosomal assembly, suggesting suppressed protein synthesis (Jirström et al., 2025). In 2024, Loher et al. (2025) performed analysis on short RNA-seq datasets with accession number GSE168714 and GSE148097 obtained from NIH’s Gene Expression Omnibus (GEO) (Dobrowolny et al., 2021; Magen et al., 2021). Both datasets consist of serum and plasma samples from ALS patients and control group. From the analysis, they found that the abundance of tRF-5 and tRF-3 and a few 5′-tiRNAs changed. In general, they re-examined the plasma samples of GEO project GSE168714 which include 103 datasets from control and 248 datasets from ALS patients using DESeq2. They identified differentially abundant 56 tRFs. Additionally, they carried out an independent analysis on the serum samples of GEO project GSE148097, containing six control and 13 patient datasets using DESeq2 and showed that 34 tRFs were differentially abundant. More specifically, the abundance of 5′-tRNA half from tRNA-Gly-GCC in ALS patients increased compared to control (Loher et al., 2025).

### 5.4 Huntington’s disease

Huntington’s disease (HD) is a neurodegenerative autosomal inherit disorder caused by the CAG repeat expansion in the huntingtin gene (HTT) (Creus-Muncunill et al., 2021; Zhao et al., 2025). The clinical features of HD are unwanted choreatic movements, behavioral and psychiatric impairments and dementia (Roos, 2010). In HD, repeat expansion resulted in aggregation of HTT protein (Kim et al., 2021). The accumulation of aggregated HTT protein primarily affects the striatum is the primarily affected region where loss of medium spiny neurons (MSN) occurs and inflammation. Although the genetic mutation that is responsible for HD has been discovered, further research is required to shed light on the pathophysiological mechanisms by which mutant HTT contributes to the disease (Pellegrini et al., 2022). Therefore, effective treatments or valid biomarkers have not been developed. In vivo and clinical studies demonstrated that there are widespread alterations at the transcriptional level primarily in the striatum (Dubois et al., 2021). Previously, the association of ncRNAs including miRNAs and long non-coding RNAs (lncRNAs) with pathophysiology of HD has been demonstrated (Tan et al., 2021; Ghafouri-Fard et al., 2022).

The role of tsRNAs in HD has recently emerged as a field of study. In one study, small RNAs derived from the putamen of HD patients (HD-sRNA-PT) were introduced into the striatum of mice, leading to the recapitulation of neuropathological features (Creus-Muncunill et al., 2021). The small RNAs in mice injected with HD-sRNA-PT were characterized by small RNA sequencing. The injection of HD-sRNA-PT into mice resulted in the abundance of tRNA clusters. In HD-sRNA-PT injected mice, Ala-, Gly- and Val-5′ tRFs were upregulated particularly in putamen. The selected 5′ tRFs were overexpressed in primary striatum neurons of mice and their effect on neuronal viability was assessed. According to MTS assay, overexpression of 5′ tRF derived from tRNA-Ala-CGC-3 was sufficient to reduce cell viability. tRNA-Ala-CGC-3 is a target of the Dopamine- and cAMP-regulated phosphoprotein (DARPP-32) protein, a critical player in Huntington’s disease pathogenesis, and functional studies using its inhibitor have validated this interaction.

### 5.5 Multiple sclerosis

Multiple sclerosis (MS) is an inflammatory-mediated ND of the CNS defined by inflammation, demyelination, and neurodegeneration. MS is a multifactorial disease that is determined by genetic (e.g., genetic variants in HLA complex), epigenetic and environmental factors (vitamin D deficiency, Epstein-Barr virus (EBV) infection and microbiome) (Maciak et al., 2021; Wang, 2021). Most of the people are diagnosed with relapsing–remitting multiple sclerosis (RRMS) which is distinguished by having periods of worsening and followed by a relative stabilization until the next attack (Duffy and McCoy, 2020; Manu et al., 2021). After 10–15 years, the diagnosis of RRMS, patients developed secondary progressive multiple sclerosis (SPMS) leading to chronic inflammation and increasing neurological disability (Yousuf and Qurashi, 2021). The rare type of MS is primary progressive multiple sclerosis (PPMS), symptoms worsen after the initial diagnosis and periods of relapse–remission cannot be observed. The occurrence of different phenotypes is a consequence of the complexity of MS. For the development of MS subtype-specific therapies, it is essential to identify biomarkers in serum, plasma, or CSF that can differentiate between the various subtypes of multiple sclerosis.

The diagnostic potential of various miRNAs and lncRNAs has been previously established (Nowak et al., 2022; Alkhazaali-Ali et al., 2024). According to the current literature, there is only one article that has explored the role of tsRNAs in MS (Needhamsen et al., 2022). In this study, small RNA sequencing was performed on blood and CSF samples of MS patients. The result of MINTmap pipeline indicated that tRF-3constitute the majority of transcripts detected in plasma samples. The most abundantly detected tsRNAs in CSF and CSF cells were i-tRFs and tRF-5, respectively. They detected tRF-36-PJB7MNLE308HP1B was significantly upregulated in plasma samples of RRMS patients.

### 5.6 Ischemic stroke

Ischemic injuries occur as a result of disrupted or inadequate blood flow, potentially resulting in functional impairment of affected organs. Ischemic Stroke (IS) is a significant cause of disability and mortality in adults, stemming from cerebrovascular events. Following an IS, the conditions of ischemia and anoxia contribute to neuronal degeneration and necrosis (Lou et al., 2012). Limited research has explored the role of tsRNAs in ischemia, but a rat model of ischemia induced by middle cerebral artery occlusion (MCAO) revealed tRNA-Val and tRNA-Gly fragments as the most prevalent small RNA fragments (Li et al., 2016). In PC12 cells, exposure to arsenite or hydrogen peroxide triggered oxidative stress, leading to tRNA cleavage and tiRNA production. An *in vitro* ischemia–reperfusion injury model in PC12 cells showed that under stress conditions, ANG-mediated generation of tiRNAs, including tRNA-Ala, tRNA-Gly, and tRNA-Cys, was induced, as observed through Northern blotting. Minocycline treatment in an ischemia–reperfusion PC12 cell model mitigated oxygen-glucose-deprivation (OGD)-induced damage by significantly down-regulating tiRNA biomarkers linked to cell injury (Elkordy et al., 2018). Excessive glutamate release leading to neuronal necrosis is a well-established contributor to morbidity and mortality in IS. To investigate the underlying mechanisms, small RNA sequencing was performed in glutamate-induced rat primary neurons and found 82 tRFs and tiRNA were upregulated and 71 tRFs and tiRNA were downregulated. Six tRFs and tiRNA, which were upregulated in the glutamate treated group were tested for their cytotoxic effect. tRF-His-GTG and tRF-Gln-CTG showed stronger cytotoxic effect upon transfection, triggered cell swelling and death. Members of the tRF-2 subclass, including tRF-His-GTG and tRF-Gln-CTG, demonstrated novel cytotoxic properties, indicating that while certain tiRNAs may promote neuronal vulnerability and progressive degeneration, specific tRF-2 species act as potent inducers of acute neuronal death. These tRFs impaired protein synthesis and mitochondrial function which confirmed by functional experiments in both in vitro and Drosophila models (Cao et al., 2021). In a rat model of cerebral ischemia/reperfusion injury, minocycline treatment reduced tiRNA levels and induced selective tRNA cleavage, indicating modulation of tiRNA generation (Sato et al., 2020). In chronic-phase intracerebral hemorrhage in rats, 331 tsRNAs were identified, with seven significantly altered (one upregulated, six downregulated). Bioinformatics and validation linked these tsRNAs to oxidative stress, endocytosis, and GPCR signaling, implicating them in hemorrhagic stroke pathology (Li et al., 2020).

A clinical study found elevated plasma tRNA derivatives in both ischemic and hemorrhagic stroke patients versus controls. In IS, higher levels correlated with larger infarcts, greater hematoma volumes, and poorer 7-day outcomes, suggesting their potential as early biomarkers (Ishida et al., 2020). Blood sample analysis two days post-stroke identified six significantly upregulated tRFs and highlighted CD14+ monocytes as central players in the cholinergic inflammatory reflex, linking specific tRFs to immune responses following stroke (Winek et al., 2020). Small RNA sequencing of plasma samples from a discovery cohort of acute IS, intracerebral hemorrhage (ICH), and stroke mimics (SM) patients revealed that tRFs were most abundant in ICH plasma. The analysis demonstrated high diagnostic accuracy for differentiating ICH from IS and SM, with tRFs showing promise as biomarkers for distinguishing between these stroke types and controls, with validation yielding strong results in independent datasets (Nguyen et al., 2020). In a study of 17 patients with acute IS due to anterior large vessel occlusion, elevated plasma tRNA derivatives correlated with infarct size and outcomes, with lower levels predicting better prognosis after endovascular thrombectomy, suggesting their potential as early biomarker (Ishida et al., 2022).

### 5.7 Epilepsy

Epilepsy is a neurological disorder defined by the repeated seizures (Beghi et al., 2015). Diagnosis of epilepsy relies on the patient’s clinical history, along with Electroencephalogram (EEG) and Magnetic Resonance Imaging (MRI) scans. However, EEG based seizure prediction studies often lack accuracy for some patients. Blood-based biomarkers, such as ncRNAs (like tRFs) are considered as a promising alternative due to their ease of collection and rapid analysis. It has been shown that three tRFs (5′ Gly-GCC, 5′ Ala-TGC, and 5′ Glu-CTC) obtained from plasma samples of patients with focal epilepsy during video EEG monitoring are significantly different compared to those in healthy controls. These fragments were found at higher levels before seizures than after, suggesting their potential as biomarkers for assessing seizure risk (Hogg et al., 2019).

### 5.8 Glioma

Glioma is a common malignant tumor that originates primarily in the brain and other areas of the central nervous system, typically developing from glial cells. In 2007, World Health Organization (WHO) classified CNS tumors into four categories from grade 1 to 4. According to this classification, glioblastoma multiforme (GBM) is graded as the most severe grade 4 tumor and patients with GBM show poor clinical outcome (Chen et al., 2017; Yasinjan et al., 2023). Despite advances in treatment, there is a critical need for the deeper understanding of the underlying molecular mechanisms to improve diagnostic and prognostic tools. tRFs as regulatory molecules could provide new insights into glioma biology and clinical management.

In previous study, researchers explored the differences in tsRNA expression in patients with GBM (4 cases) and low-grade glioma (LG) (five cases). RNA sequencing from the fresh tumor samples showed differences in the expression of tsRNAs, and among nine candidate tsRNAs, three tsRNAs showed low expression and six tsRNAs showed high expression in GBMs. Based on quantitative PCR (qPCR), the expression of one tsRNA (tRF-1–32-chrM.Lys-TTT) was significantly decreased in GBM compared to LG. On the other hand, the expressions of 5 tsRNAs were significantly increased. Gene ontology enrichment analysis indicated these tsRNAs are associated with nucleotide excision repair (NER), Hippo signaling pathways, and other cancer-related processes (Tu et al., 2023). Complementing these findings, Ren et al. (2022) conducted comprehensive bioinformatics and experimental studies. They analyzed over 1,000 human tsRNAs from tRFexplorer and tRFdb and mapped them back to their original tRNA sources. They found that about 200 tsRNAs were actively expressed in glioma samples and focused primarly on tRNA-Cys-GCA. tRFs derived from tRNA-Cys-GCA, can directly regulate oncogenes like VAV2, thereby influencing proliferation, autophagy, and survival outcomes. It produced nine different tsRNA fragments from various regions including four tRF-5 (tRFdb-5015a, tRFdb-5016a, tRFdb-5017a, and tRFdb-5017b), two tRF-3 (tRFdb-3003a and tRFdb-3003b) and three tRF-1 (ts-55, ts-56, and ts-60). Among them the expression of tRFdb-3003a/b, ts-55, and ts-60 were decreased in glioma compared to non-tumors. They also investigated the association between the expression of tRFdb-3003a and tRFdb-3003b and survival outcome of the patients by Kaplan–Meier curve analyses. They indicated that downregulation of these two tsRNAs were linked to poor survival. Moreover, they suggested that tRFdb-3003a may involve in regulation of glioma cell proliferation *in vivo* and *in vitro*. Analysis revealed that mRNAs correlated with tRFdb-3003a were enriched in pathways related to autophagy and the spliceosome. These findings suggest that the reduced expression of tRFdb-3003a may contribute to glioma progression and could function as a potential tumor suppressor (Ren et al., 2022).

To bridge computational findings with the mechanistic understanding of glioma, researchers investigated TRMT10A expression, whose low expression is associated with poorer prognosis in GBM patients. They found that knockdown of TRMT10A expression in U251 glioma cells resulted in upregulation of tRNA-Arg-CCT-derived tRF-22 (tRF-22-8XF6RE98N) along with decrease in m1G9 modification of tRNA-Arg-CCT. In contrast, tRF-22 inhibition coupled with TRMT10A overexpression significantly diminished xenograft tumor size and vasculogenic mimicry in nude mice (Wei et al., 2025).

Extending mechanistic investigations to explore the presence of tRFs in microvesicles and exosomes, Gyuris et al. (2019) isolated cancer EVs and ribonucleoprotein complexes from mouse primary GBM cells at were driven by specific growth factor receptors (EGFR and PDGFRA). They performed RNA sequencing to determine long and short RNAs and discovered that ribonucleoprotein complexes contain high concentrations of tRFs. Their study indicated the possible role of tRFs in how cells communicate with each other (Gyuris et al., 2019).

### 5.9 Major depressive disorder

Depression is a mood disorder characterized by a persistent feeling of sadness and loss of interest, affecting an individual’s emotions, thoughts, and behaviors, and potentially leading to various emotional and physical problems. This condition places considerable health and social challenges worldwide, as evidenced by extensive research (Li et al., 2023). To understand the role of tsRNAs in depression-related disorders, researchers analyzed sncRNAs from the prefrontal cortex of 93 individuals with schizophrenia or bipolar disorder and 77 controls. Distinct profiles of sncRNAs were identified, with a notable abundance of tRNA-derived fragments, particularly 5′ tiRNAs from tRNA-Glu-CTC and tRNA-Gly-CCC/Gly-GCC, as the most commonly expressed in both groups (Nersisyan et al., 2024). In a follow-up study involving 258 patients with major depressive disorder (MDD), small RNA sequencing was used to assess tsRNA expression before and after 8 weeks of duloxetine treatment. Ten tsRNAs showed significant expression changes in responders and were found to interact with ECM1, BAFF, and miRNAs, suggesting their involvement in the drug’s therapeutic effects through modulation of key biological pathways (Wang et al., 2025). In a chronic unpredictable mild stress (CUMS)-induced depression mouse model, 14 tsRNAs were differentially expressed, and Fer-1 treatment altered 22 tsRNAs (seven upregulated, 15 downregulated). Notably, tsRNA-3029b, upregulated in CUMS and downregulated by Fer-1, reduced ferroptosis and promoted neuronal regeneration when inhibited, suggesting that Fer-1’s antidepressant effects may involve tsRNA modulation (Li et al., 2023). Also, CUMS-induced depressive like mouse model used to investigate how psychological stress influences male fertility and alters sperm small non-coding RNAs. Notably, significant alterations in sperm tsRNAs were observed, with 988 downregulated and 26 upregulated, particularly affecting tsRNA-Leu and tsRNA-Gly types, suggesting a potential link to stress-related fertility decline (Huang et al., 2025).

### 5.10 Autism spectrum disorder

Autism spectrum disorder (ASD) is a neurodevelopmental condition which is defined by impairment in social communication along with repetitive behaviors and focused interests (Wu et al., 2016). While existing diagnostic approaches can determine ASD in children by approximately age three, early detection remains challenging and may lead to a significant number of false positive results (Hicks et al., 2016). Maternal immune activation (MIA) during pregnancy have been identified as potential ASD risk factor which may influence fetal development by altering epigenetic processes.

Recent studies suggest that MIA alters the expression of miRs and tRFs at the maternal-fetal interface. In one study, researchers collected RNA from the placenta/decidua of pregnant mice at day E12.5 after administering a second dose of poly (I:C) which is known to trigger autism-related traits in MIA offspring. Afterward, they performed RNA sequencing on the extracted samples. MIA temporarily led to a significant increase in the expression of seven tRF-3a variants which is followed by normalization of the expression within 6 h. On the other hand, 5’ tiRNAs from tRNA-Asp showed significant downregulation following MIA at 3 h and exhibited decreased expression at 6 h. This study showed the role of tRFs in the regulation of neurodevelopment and suggested potential for biomarker-based early screening of ASD (Su et al., 2020a).

### 5.11 Traumatic brain injury

Traumatic brain injury (TBI) is defined by a complicated pathophysiology that initiates with a primary mechanical insult and advances into an extended secondary injury phase, which encompasses various interconnected pathological mechanisms. Gaining insight into the functional roles of small non-coding RNAs (sncRNAs) could offer new perspectives and therapeutic approaches for tackling the intricate nature of brain disorders, including TBI (Puhakka et al., 2022).

In a mouse model of controlled cortical impact, 103 tsRNAs were differentially expressed 72 h after traumatic brain injury (TBI), with 56 upregulated and 47 downregulated. Gene ontology analysis indicated involvement in inflammation and synaptic activity, suggesting tsRNAs may contribute to secondary injury and serve as potential therapeutic targets (Xu et al., 2022). Three months post-TBI, small RNA sequencing in rats with chronic neuroinflammation revealed altered small non-coding RNA profiles, including 16 and 13 tRFs in the thalamus and cortex, respectively. Two upregulated fragments, 3′ tRF-Ile-AAT and 3′ tRF-Lys-TTT, were linked to worse behavioral outcomes, suggesting a regulatory role for tsRNAs in chronic neuroinflammation and their potential as therapeutic targets (Puhakka et al., 2022). Xuefu Zhuyu Decoction (XFZYD), a traditional Chinese medicine, modulated 41 specific tsRNAs in TBI rats, suggesting tsRNA involvement in key recovery pathways and potential therapeutic effects (Yang et al., 2022). Sequencing and omics analyses in a TBI rat model identified XFZYD-regulated tsRNAs, notably AS-tDR-002004 and AS-tDR-002583, as potential therapeutic targets influencing key injury-related pathways via genes such as *Pi4kb*, *Mapk1*, and *Gnai1* (Dai et al., 2022).

### 5.12 Spinal cord injury

Spinal cord injury (SCI) primarily results from traumatic events, starting with immediate physical damage from sudden compression and bruising of the spinal cord. This initial injury triggers a cascade of secondary biological processes which usually lead to a permanent neurological impairment (Qin et al., 2019). Numerous treatment approaches have been developed to minimize further neuronal damage counteract neurodegeneration while enhancing neural repair and clinical improvement (Anjum et al., 2020). Although some progress has been made, complete therapeutic breakthrough remains challenging. Therefore, the mechanisms underlying SCI need to be understood better and new molecular targets for therapeutic applications must be identified. A study explored tsRNA as potential mediators of the pathophysiological modifications observed after spinal cord injury. Researchers used tsRNA-Seq analysis to determine the tsRNA expression profiles in spinal cord tissues from sham and contusion SCI rat models. The results revealed that a total of 47 tsRNAs were exclusively expressed in the sham group and 28 tsRNAs were exclusively expressed in SCI group. In general, tRF-5 expression was upregulated in SCI group while other tsRNA types were downregulated. Additionally, tiRNA-Gly-GCC-001 was found to be significantly upregulated in the SCI group by both sequencing and PCR. This study also demonstrated that tiRNA-Gly-GCC-001 suppressed BDNF expression through direct binding to its 3′ UTR. Through targeting BDNF, it could modulate MAPK and neurotrophin signaling pathways, thus influencing pathophysiological changes following SCI (Qin et al., 2019).

## 6 Biomarker and therapeutic potential of tRFs

Dysregulation of tRFs level is linked to several neuropsychiatric disorders. Advances in RNA-seq technologies and bioinformatics have revealed tissue-specific expression patterns of tsRNAs, highlighting their potential as biomarkers. Their stability and detectability in CSF, serum, and brain tissue, along with disease- and regional expression pattern and robust changes in response to various stimuli, suggest their suitability for clinical use as non-invasive biomarkers; as shown in studies differentiating PD patients from controls (Godoy et al., 2018; Magee et al., 2019). Additionally, their presence in EVs provides an accessible sample source that reflects changes occurring in the brain (Torres and Martí, 2021). Together, these characteristics make tRFs ideal candidates for diagnostic, prognostic, and disease-monitoring biomarkers in neuropsychiatric disorders. tRFs have also emerged as novel promising therapeutic agents due to their regulatory roles in various cellular process. However, before clinical implementation, the roles of tsRNAs in disease mechanisms must be clarified, and their therapeutic applications carefully validated. Strategies such as overexpression, knockout models, and genome-editing approaches (e.g., CRISPR/Cas9) may help elucidate tsRNA functions and optimize therapeutic interventions. In addition, safe and efficient delivery systems, including AAV or other viral vectors, will be required to overcome obstacles such as blood–brain barrier penetration and off-target effects. Although significant hurdles persist in production, purification, and delivery, ongoing research strongly supports the feasibility of developing tsRNAs into biopharmaceuticals for treating NDs in the future (Tian et al., 2022).

The interpretation of tRFs as biomarkers must consider the heterogeneity of sample sources. Each sample type may provide unique diagnostic or mechanistic insights about the disease. For example, researchers showed that different sets of tRFs are expressed in CSF compared to blood in PD patients which separates from controls (Paldor et al., 2023). Another study supports these finding stating that most of the tRFs that change in PD compared to healthy controls are specific to only one type of sample (prefrontal cortex, CSF, or serum). Moreover, the same study demonstrated clear sex-dependent patterns. Differentially abundant tRFs in PD were observed when samples were analyzed separately by sex particularly in CSF and serum (Magee et al., 2019). Similarly, researchers showed differences in tRF expression profiles in the nucleus accumbens of individuals with AD in a sex-specific analysis of sncRNA profiles by small RNA-Seq. They detected significant alterations exclusively in females, while prominent changes were not observed in males (Shulman et al., 2023). These studies emphasize that sex is a key factor of tRF alterations and must be taken into account as a critical factors in PD and AD for future biomarker studies.

Standardization in the tRFs is important to keep reproducibility and comparability across different studies. Currently, there are substantial variations in RNA isolation methods, sequencing platforms, and bioinformatic pipelines. Additionally, a uniform nomenclature system and tools such as tDRnamer is required for consistent naming of tRFs (Holmes et al., 2023). Integration of standard protocols for sample preparation, library construction, and data analysis will help cross study comparisons while improving the reliability of tRF research. This will ultimately promote the conversion of tRFs into robust biomarkers and therapeutic targets.

## 7 Future perspective and conclusion

In recent years, tsRNAs have gained recognition as key regulatory molecules involved in the pathogenesis of neurodegenerative and psychiatric disorders. Although high-throughput sequencing has identified numerous tsRNAs associated with NDs, their biogenesis, classification, and molecular roles remain only partially understood. Emerging evidence indicates that tsRNAs are integral to CNS development and function, where they may exert neuroprotective effects and regulate neuronal gene expression. Aberrant tsRNA expression has been linked to the onset and progression of NDs, in part by modulating mRNAs encoding proteins critical for neuronal survival. This suggests their potential utility as diagnostic biomarkers and therapeutic targets. Despite their promise, clinical translation of tsRNAs is still at an early stage. The therapeutic use of tsRNAs is still confined to the preclinical stage, largely because of significant hurdles related to their delivery, stability, and safety within the CNS. Major limitations involve insufficient ability to cross the blood–brain barrier, susceptibility to rapid degradation, potential off-target interactions, and the absence of well-established strategies for reliable and safe delivery to the brain (Tian et al., 2022). Current detection methods include sequencing, microarrays, qRT-PCR, northern blotting, and computational analyses, yet more refined and integrated approaches are needed for clinical application. Furthermore, the interaction networks between tsRNAs and other non-coding RNAs, particularly miRNAs, are not yet fully delineated.

In conclusion, tsRNAs represent a novel and promising class of regulatory RNAs with potential to enhance our understanding of neurodegenerative mechanisms and contribute to the development of innovative therapeutic strategies.

## References

[B1] Aalto A. P. Pasquinelli A. E. (2012). Small non-coding RNAs mount a silent revolution in gene expression. *Curr. Opin. Cell. Biol.* 24 333–340. 10.1016/j.ceb.2012.03.006 22464106 PMC3372702

[B2] Abbasi-Moheb L. Mertel S. Gonsior M. Nouri-Vahid L. Kahrizi K. Cirak S. (2012). Mutations in NSUN2 cause autosomal-recessive intellectual disability. *Am. J. Hum. Genet.* 90 847–855. 10.1016/j.ajhg.2012.03.021 22541559 PMC3376487

[B3] Ajmeriya S. Bharti D. R. Kumar A. Rana S. Singh H. Karmakar S. (2024). In silico approach for the identification of tRNA-derived small non-coding RNAs in SARS-CoV infection. *J. Appl. Genet.* 65 403–413. 10.1007/s13353-024-00853-4 38514586

[B4] Alkhazaali-Ali Z. Sahab-Negah S. Boroumand A. R. Tavakol-Afshari J. (2024). MicroRNA (miRNA) as a biomarker for diagnosis, prognosis, and therapeutics molecules in neurodegenerative disease. *Biomed. Pharmacother.* 177:116899. 10.1016/j.biopha.2024.116899 38889636

[B5] Anderson P. Ivanov P. (2014). tRNA fragments in human health and disease. *FEBS Lett.* 588 4297–4304. 10.1016/j.febslet.2014.09.001 25220675 PMC4339185

[B6] Anjum A. Yazid M. D. Fauzi Daud M. Idris J. Ng A. M. H. Selvi Naicker A. (2020). Spinal cord injury: Pathophysiology, multimolecular interactions, and underlying recovery mechanisms. *Int. J. Mol. Sci.* 21:7533. 10.3390/ijms21207533 33066029 PMC7589539

[B7] Aparicio-Erriu I. M. Prehn J. H. M. (2012). Molecular mechanisms in amyotrophic lateral sclerosis: The role of angiogenin, a secreted RNase. *Front. Neurosci.* 6:167. 10.3389/fnins.2012.00167 23181008 PMC3500830

[B8] Arioz B. I. Binokay L. Tastan B. Genc B. Cotuk A. Dursun E. (2025). Characterization of tRNA-derived fragments in the small neuron-derived extracellular vesicles of Alzheimer’s disease patients. *Brain Res.* 1862:149730. 10.1016/j.brainres.2025.149730 40436234

[B9] Ashrafian H. Zadeh E. H. Khan R. H. (2021). Review on Alzheimer’s disease: Inhibition of amyloid beta and tau tangle formation. *Int. J. Biol. Macromol.* 167 382–394. 10.1016/j.ijbiomac.2020.11.192 33278431

[B10] Baindoor S. Gibriel H. A. Y. Venø M. T. Su J. Morrissey E. P. Jirström E. (2024). Distinct fingerprints of tRNA-derived small non-coding RNA in animal models of neurodegeneration. *Dis. Model. Mech.* 17:dmm050870. 10.1242/dmm.050870 39552337 PMC11603119

[B11] Bastide A. Yewdell J. W. David A. (2018). The RiboPuromycylation method (RPM): An Immunofluorescence technique to map translation sites at the sub-cellular level. *Bio Protoc.* 8:e2669. 10.21769/BioProtoc.2669 29552591 PMC5856242

[B12] Beghi E. Giussani G. Sander J. W. (2015). The natural history and prognosis of epilepsy. *Epileptic Disord.* 17 243–253. 10.1684/epd.2015.0751 26234761

[B13] Blanco S. Dietmann S. Flores J. V. Hussain S. Kutter C. Humphreys P. (2014). Aberrant methylation of tRNAs links cellular stress to neuro-developmental disorders. *EMBO J.* 33 2020–2039. 10.15252/embj.201489282 25063673 PMC4195770

[B14] Blaze J. Navickas A. Phillips H. L. Heissel S. Plaza-Jennings A. Miglani S. (2021). Neuronal Nsun2 deficiency produces tRNA epitranscriptomic alterations and proteomic shifts impacting synaptic signaling and behavior. *Nat. Commun.* 12:4913. 10.1038/s41467-021-24969-x 34389722 PMC8363735

[B15] Boskovic A. Bing X. Y. Kaymak E. Rando O. J. (2020). Control of noncoding RNA production and histone levels by a 5’ tRNA fragment. *Genes Dev.* 34 118–131. 10.1101/gad.332783.119 31831626 PMC6938667

[B16] Cai J. Li C. Liu S. Tan M. Sun Y. Sun X. (2024). Angiogenin-mediated tsRNAs control inflammation and metabolic disorder by regulating NLRP3 inflammasome. *Cell Death Differ.* 31 1057–1069. 10.1038/s41418-024-01311-8 38740959 PMC11303556

[B17] Cao Y. Liu K. Xiong Y. Zhao C. Liu L. (2021). Increased expression of fragmented tRNA promoted neuronal necrosis. *Cell Death Dis.* 12:823. 10.1038/s41419-021-04108-6 34462418 PMC8405691

[B18] Chan P. P. Holmes A. D. Lowe T. M. (2025). Analyzing, visualizing, and annotating tRNA-derived RNAs using tRAX and tDRnamer. *Methods Enzymol.* 711 103–133. 10.1016/bs.mie.2024.11.016 39952700 PMC12503770

[B19] Chen L. Liu B. (2017). Relationships between stress granules, oxidative stress, and neurodegenerative diseases. *Oxid. Med. Cell. Longev.* 2017:1809592. 10.1155/2017/1809592 28194255 PMC5286466

[B20] Chen R. Smith-Cohn M. Cohen A. L. Colman H. (2017). Glioma subclassifications and their clinical significance. *Neurotherapeutics* 14 284–297. 10.1007/s13311-017-0519-x 28281173 PMC5398991

[B21] Chu X. He C. Sang B. Yang C. Yin C. Ji M. (2022). Transfer RNAs-derived small RNAs and their application potential in multiple diseases. *Front. Cell. Dev. Biol.* 10:954431. 10.3389/fcell.2022.954431 36072340 PMC9441921

[B22] Cole C. Sobala A. Lu C. Thatcher S. R. Bowman A. Brown J. W. S. (2009). Filtering of deep sequencing data reveals the existence of abundant Dicer-dependent small RNAs derived from tRNAs. *RNA* 15 2147–2160. 10.1261/rna.1738409 19850906 PMC2779667

[B23] Cooke W. R. Jiang P. Ji L. Bai J. Jones G. D. Lo Y. M. D. (2024). Differential 5’-tRNA fragment expression in circulating preeclampsia syncytiotrophoblast vesicles drives macrophage inflammation. *Hypertension* 81 876–886. 10.1161/HYPERTENSIONAHA.123.22292 38362745 PMC10956686

[B24] Creus-Muncunill J. Guisado-Corcoll A. Venturi V. Pantano L. Escaramís G. García (2021). Huntington’s disease brain-derived small RNAs recapitulate associated neuropathology in mice. *Acta Neuropathol.* 141 565–584. 10.1007/s00401-021-02272-9 33547932

[B25] Dai F. Tang T. Lu R. Li P. Feng D. Hu M. (2022). Systematic analysis of tRNA-derived small RNAs reveals the effects of Xuefu-Zhuyu decoction on the hippocampi of rats after traumatic brain injury. *Evid. Based Complement. Alternat. Med.* 2022:5748719. 10.1155/2022/5748719 36164400 PMC9509243

[B26] Deng L. Wang H. Fan T. Chen L. Shi Z. Mi J. (2022). Potential functions of the tRNA-derived fragment tRF-Gly-GCC associated with oxidative stress in radiation-induced lung injury. *Dose Response* 20:15593258221128744. 10.1177/15593258221128744 36176737 PMC9513591

[B27] Deng Z. Li Y. Chi W. Zhang W. Li F. Ling L. (2025). tRFAla-AGC-3-M8 attenuates neuroinflammation and neuronal damage in Alzheimer’s disease via the EphA7-ERK1/2-p70S6K signaling pathway. *Alzheimers Res. Ther.* 17:104. 10.1186/s13195-025-01734-6 40375351 PMC12079980

[B28] Do A. N. Magesh S. Uzelac M. Chen T. Li W. T. Bouvet M. (2024). Computational analysis suggests that AsnGTT 3′-tRNA-derived fragments are potential biomarkers in papillary thyroid carcinoma. *Int. J. Mol. Sci.* 25:10631. 10.3390/ijms251910631 39408960 PMC11476591

[B29] Dobin A. Davis C. A. Schlesinger F. Drenkow J. Zaleski C. Jha S. (2013). STAR: Ultrafast universal RNA-seq aligner. *Bioinformatics* 29 15–21. 10.1093/bioinformatics/bts635 23104886 PMC3530905

[B30] Dobrowolny G. Martone J. Lepore E. Casola I. Petrucci A. Inghilleri M. (2021). A longitudinal study defined circulating microRNAs as reliable biomarkers for disease prognosis and progression in ALS human patients. *Cell Death Discov.* 7:4. 10.1038/s41420-020-00397-6 33431881 PMC7801652

[B31] Dong X. Li Q. Li R. Li Y. Jin F. Li H. (2025). Inhibition of tRF- 02514 in extracellular vesicles preserves microglia pyroptosis and protects against Parkinson’s disease. *Mol. Neurobiol*. 62 11047–11063. 10.1007/s12035-025-04925-2 40254704 PMC12367836

[B32] Donovan P. D. McHale N. M. Venø M. T. Prehn J. H. M. (2021). tsRNAsearch: A pipeline for the identification of tRNA and ncRNA fragments from small RNA-sequencing data. *Bioinformatics* 37 4424–4430. 10.1093/bioinformatics/btab515 34255836

[B33] Dubnov S. Bennett E. R. Yayon N. Yakov O. Bennett D. A. Seshadri S. (2024). Knockout of the longevity gene Klotho perturbs aging and Alzheimer’s disease-linked brain microRNAs and tRNA fragments. *Commun. Biol.* 7:720. 10.1038/s42003-024-06407-y 38862813 PMC11166644

[B34] Dubois C. Kong G. Tran H. Li S. Pang T. Y. Hannan A. J. (2021). Small non-coding RNAs are dysregulated in Huntington’s disease transgenic mice independently of the therapeutic effects of an environmental intervention. *Mol. Neurobiol.* 58 3308–3318. 10.1007/s12035-021-02342-9 33675499

[B35] Duffy C. P. McCoy C. E. (2020). The role of MicroRNAs in repair processes in multiple sclerosis. *Cells* 9:1711. 10.3390/cells9071711 32708794 PMC7408558

[B36] Elkordy A. Mishima E. Niizuma K. Akiyama Y. Fujimura M. Tominaga T. (2018). Stress-induced tRNA cleavage and tiRNA generation in rat neuronal PC12 cells. *J. Neurochem.* 146 560–569. 10.1111/jnc.14321 29431851

[B37] Emara M. M. Ivanov P. Hickman T. Dawra N. Tisdale S. Kedersha N. (2010). Angiogenin-induced tRNA-derived stress-induced RNAs promote stress-induced stress granule assembly. *J. Biol. Chem.* 285 10959–10968. 10.1074/jbc.M109.077560 20129916 PMC2856301

[B38] Esmaeili F. Banerjee K. Su Z. Dutta A. (2025). A general framework to over-express tRNA-derived fragments from their parental tRNAs in mammalian cells. *Methods Enzymol.* 711 241–259. 10.1016/bs.mie.2024.11.008 39952708 PMC12020451

[B39] Flores J. V. Cordero-Espinoza L. Oeztuerk-Winder F. Andersson-Rolf A. Selmi T. Blanco S. (2017). Cytosine-5 RNA methylation regulates neural stem cell differentiation and motility. *Stem Cell Rep.* 8 112–124. 10.1016/j.stemcr.2016.11.014 28041877 PMC5233436

[B40] Fu H. Feng J. Liu Q. Sun F. Tie Y. Zhu J. (2009). Stress induces tRNA cleavage by angiogenin in mammalian cells. *FEBS Lett.* 583 437–442. 10.1016/j.febslet.2008.12.043 19114040

[B41] Gagliardi S. Davin A. Bini P. Sinforiani E. Poloni T. E. Polito L. (2019). A novel nonsense angiogenin mutation is associated with Alzheimer disease. *Alzheimer Dis. Assoc. Disord.* 33 163–165. 10.1097/WAD.0000000000000272 30188356

[B42] George S. Rafi M. Aldarmaki M. ElSiddig M. Al Nuaimi M. Amiri K. M. A. (2022). tRNA derived small RNAs-Small players with big roles. *Front. Genet.* 13:997780. 10.3389/fgene.2022.997780 36199575 PMC9527309

[B43] Ghafouri-Fard S. Khoshbakht T. Hussen B. M. Taheri M. Ebrahimzadeh K. Noroozi R. (2022). The emerging role of long non-coding RNAs, microRNAs, and an accelerated epigenetic age in Huntington’s disease. *Front. Aging Neurosci.* 14:987174. 10.3389/fnagi.2022.987174 36185471 PMC9520620

[B44] Godoy P. M. Bhakta N. R. Barczak A. J. Cakmak H. Fisher S. MacKenzie T. C. (2018). Large differences in small RNA composition between human biofluids. *Cell Rep.* 25 1346–1358. 10.1016/j.celrep.2018.10.014 30380423 PMC6261476

[B45] Goodarzi H. Liu X. Nguyen H. C. B. Zhang S. Fish L. Tavazoie S. F. (2015). Endogenous tRNA-Derived fragments suppress breast cancer progression via YBX1 displacement. *Cell* 161 790–802. 10.1016/j.cell.2015.02.053 25957686 PMC4457382

[B46] Green J. A. Ansari M. Y. Ball H. C. Haqqi T. M. (2020). tRNA-derived fragments (tRFs) regulate post-transcriptional gene expression via AGO-dependent mechanism in IL-1β stimulated chondrocytes. *Osteoarthritis Cartilage* 28 1102–1110. 10.1016/j.joca.2020.04.014 32407895 PMC8418333

[B47] Greenway M. J. Andersen P. M. Russ C. Ennis S. Cashman S. Donaghy C. (2006). ANG mutations segregate with familial and “sporadic” amyotrophic lateral sclerosis. *Nat. Genet.* 38 411–413. 10.1038/ng1742 16501576

[B48] Gyuris A. Navarrete-Perea J. Jo A. Cristea S. Zhou S. Fraser K. (2019). Physical and molecular landscapes of mouse glioma extracellular vesicles define heterogeneity. *Cell. Rep.* 27 3972–3987.e6. 10.1016/j.celrep.2019.05.089 31242427 PMC6604862

[B49] Hanada T. Weitzer S. Mair B. Bernreuther C. Wainger B. J. Ichida J. (2013). CLP1 links tRNA metabolism to progressive motor-neuron loss. *Nature* 495 474–480. 10.1038/nature11923 23474986 PMC3674495

[B50] He P. Zhang B. Jiang W. Zhu F. Liang Z. Gao L. (2025). PKM2 is a key factor to regulate neurogenesis and cognition by controlling lactate homeostasis. *Stem Cell Rep.* 20:102381. 10.1016/j.stemcr.2024.11.011 39706177 PMC11784464

[B51] Hicks S. D. Ignacio C. Gentile K. Middleton F. A. (2016). Salivary miRNA profiles identify children with autism spectrum disorder, correlate with adaptive behavior, and implicate ASD candidate genes involved in neurodevelopment. *BMC Pediatr.* 16:52. 10.1186/s12887-016-0586-x 27105825 PMC4841962

[B52] Hogg M. C. Raoof R. El Naggar H. Monsefi N. Delanty N. O’Brien D. F. (2019). Elevation in plasma tRNA fragments precede seizures in human epilepsy. *J. Clin. Invest.* 129 2946–2951. 10.1172/JCI126346 31039137 PMC6597227

[B53] Hogg M. C. Rayner M. Susdalzew S. Monsefi N. Crivello M. Woods I. (2020). 5’ValCAC tRNA fragment generated as part of a protective angiogenin response provides prognostic value in amyotrophic lateral sclerosis. *Brain Commun.* 2:fcaa138. 10.1093/braincomms/fcaa138 33543130 PMC7850272

[B54] Holmes A. D. Chan P. P. Chen Q. Ivanov P. Drouard L. Polacek N. (2023). A Standardized ontology for Naming tRNA-derived RNAs based on molecular origin. *Nat. Methods* 20 627–628. 10.1038/s41592-023-01813-2 36869120 PMC10334869

[B55] Hou J. Li Q. Wang J. Lu W. (2022). tRFs and tRNA halves: Novel cellular defenders in multiple biological processes. *Curr. Issues Mol. Biol.* 44 5949–5962. 10.3390/cimb44120405 36547066 PMC9777342

[B56] Huang H.-Y. Hopper A. K. (2016). Multiple layers of stress-induced regulation in tRNA biology. *Life* 6:16. 10.3390/life6020016 27023616 PMC4931453

[B57] Huang J.-J. Zhang J. Wang T. Li X. Zhang H. Wang J. (2025). Small non-coding RNA profiles in sperms from depressive-like mice induced by chronic unpredictable mild stimulations. *J. Affect. Disord.* 376 376–385. 10.1016/j.jad.2025.02.023 39961446

[B58] Inoue M. Hada K. Shiraishi H. Yatsuka H. Fujinami H. Morisaki I. (2020). Tyrosine pre-transfer RNA fragments are linked to p53-dependent neuronal cell death via PKM2. *Biochem. Biophys. Res. Commun.* 525 726–732. 10.1016/j.bbrc.2020.02.157 32143824

[B59] Ishida T. Inoue T. Niizuma K. Inoue T. Sasaki K. Sakata H. (2022). Plasma tRNA derivatives concentrations for detecting early brain damage in patients with acute large vessel occlusion and predicting clinical outcomes after endovascular thrombectomy. *Clin. Neurol. Neurosurg.* 220:107358. 10.1016/j.clineuro.2022.107358 35802994

[B60] Ishida T. Inoue T. Niizuma K. Konno N. Suzuki C. Inoue T. (2020). Prediction of functional outcome in patients with acute stroke by measuring tRNA derivatives. *Cerebrovasc. Dis.* 49 639–646. 10.1159/000511627 33207351

[B61] Ivanov P. Emara M. M. Villen J. Gygi S. P. Anderson P. (2011). Angiogenin-induced tRNA fragments inhibit translation initiation. *Mol. Cell.* 43 613–623. 10.1016/j.molcel.2011.06.022 21855800 PMC3160621

[B62] Ivanov P. O’Day E. Emara M. M. Wagner G. Lieberman J. Anderson P. (2014). G-quadruplex structures contribute to the neuroprotective effects of angiogenin-induced tRNA fragments. *Proc. Natl. Acad. Sci. U S A.* 111 18201–18206. 10.1073/pnas.1407361111 25404306 PMC4280610

[B63] Jacovetti C. Donnelly C. Menoud V. Suleiman M. Cosentino C. Sobel J. (2024). The mitochondrial tRNA-derived fragment, mt-tRF-LeuTAA, couples mitochondrial metabolism to insulin secretion. *Mol. Metab.* 84:101955. 10.1016/j.molmet.2024.101955 38704026 PMC11112368

[B64] Jirström E. Matveeva A. Baindoor S. Donovan P. Ma Q. Morrissey E. P. (2025). Effects of ALS-associated 5’tiRNAGly-GCC on the transcriptomic and proteomic profile of primary neurons in vitro. *Exp. Neurol.* 385:115128. 10.1016/j.expneurol.2024.115128 39719207

[B65] Karaca E. Weitzer S. Pehlivan D. Shiraishi H. Gogakos T. Hanada T. (2014). Human CLP1 mutations alter tRNA biogenesis, affecting both peripheral and central nervous system function. *Cell* 157 636–650. 10.1016/j.cell.2014.02.058 24766809 PMC4146440

[B66] Karacicek B. Katkat E. Binokay L. Ozhan G. Karakülah G. Genc S. (2025). The role of tRNA fragments on neurogenesis alteration by H_2_O_2_-induced oxidative stress. *J. Mol. Neurosci.* 75:47. 10.1007/s12031-025-02330-x 40216606 PMC11991940

[B67] Karousi P. Katsaraki K. Papageorgiou S. G. Pappa V. Scorilas A. Kontos C. K. (2019). Identification of a novel tRNA-derived RNA fragment exhibiting high prognostic potential in chronic lymphocytic leukemia. *Hematol. Oncol.* 37 498–504. 10.1002/hon.2616 30945323

[B68] Khan M. A. Rafiq M. A. Noor A. Hussain S. Flores J. V. Rupp V. (2012). Mutation in NSUN2, which encodes an RNA methyltransferase, causes autosomal-recessive intellectual disability. *Am. J. Hum. Genet.* 90 856–863. 10.1016/j.ajhg.2012.03.023 22541562 PMC3376419

[B69] Kieran D. Sebastia J. Greenway M. J. King M. A. Connaughton D. Concannon C. G. (2008). Control of motoneuron survival by angiogenin. *J. Neurosci.* 28 14056–14061. 10.1523/JNEUROSCI.3399-08.2008 19109488 PMC6671464

[B70] Kim A. Lalonde K. Truesdell A. Gomes Welter P. Brocardo P. S. Rosenstock T. R. (2021). New avenues for the treatment of Huntington’s disease. *Int. J. Mol. Sci.* 22:8363. 10.3390/ijms22168363 34445070 PMC8394361

[B71] Kim D. Kim H. K. Kay M. A. (2025). Functional analysis of tRNA-derived small translational regulation. *Methods Enzymol.* 711 336–355. 10.1016/bs.mie.2024.11.017 39952714

[B72] Kim D. Paggi J. M. Park C. Bennett C. Salzberg S. L. (2019). Graph-based genome alignment and genotyping with HISAT2 and HISAT-genotype. *Nat. Biotechnol.* 37 907–915. 10.1038/s41587-019-0201-4 31375807 PMC7605509

[B73] Kim H. K. Fuchs G. Wang S. Wei W. Zhang Y. Park H. (2017). A transfer-RNA-derived small RNA regulates ribosome biogenesis. *Nature* 552 57–62. 10.1038/nature25005 29186115 PMC6066594

[B74] Kim H. K. Xu J. Chu K. Park H. Jang H. Li P. (2019). A tRNA-derived small RNA regulates ribosomal protein S28 protein levels after translation initiation in humans and Mice. *Cell Rep.* 29 3816–3824.e4. 10.1016/j.celrep.2019.11.062 31851915 PMC7451100

[B75] Kuhle B. Chen Q. Schimmel P. (2023). tRNA renovatio: Rebirth through fragmentation. *Mol. Cell.* 83 3953–3971. 10.1016/j.molcel.2023.09.016 37802077 PMC10841463

[B76] Kumar P. Anaya J. Mudunuri S. B. Dutta A. (2014). Meta-analysis of tRNA derived RNA fragments reveals that they are evolutionarily conserved and associate with AGO proteins to recognize specific RNA targets. *BMC Biol.* 12:78. 10.1186/s12915-014-0078-0 25270025 PMC4203973

[B77] Kumar P. Kuscu C. Dutta A. (2016). Biogenesis and function of transfer RNA-related fragments (tRFs). *Trends Biochem. Sci.* 41 679–689. 10.1016/j.tibs.2016.05.004 27263052 PMC5173347

[B78] Kumar P. Mudunuri S. B. Anaya J. Dutta A. (2015). tRFdb: A database for transfer RNA fragments. *Nucleic Acids Res.* 43 D141–D145. 10.1093/nar/gku1138 25392422 PMC4383946

[B79] Kuscu C. Kumar P. Kiran M. Su Z. Malik A. Dutta A. (2018). tRNA fragments (tRFs) guide Ago to regulate gene expression post-transcriptionally in a Dicer-independent manner. *RNA* 24 1093–1105. 10.1261/rna.066126.118 29844106 PMC6049499

[B80] Langmead B. Salzberg S. L. (2012). Fast gapped-read alignment with Bowtie 2. *Nat. Methods* 9 357–359. 10.1038/nmeth.1923 22388286 PMC3322381

[B81] Lee Y. S. Shibata Y. Malhotra A. Dutta A. (2009). A novel class of small RNAs: tRNA-derived RNA fragments (tRFs). *Genes Dev.* 23 2639–2649. 10.1101/gad.1837609 19933153 PMC2779758

[B82] Levitz R. Chapman D. Amitsur M. Green R. Snyder L. Kaufmann G. (1990). The optional E. coli prr locus encodes a latent form of phage T4-induced anticodon nuclease. *EMBO J.* 9 1383–1389. 10.1002/j.1460-2075.1990.tb08253.x 1691706 PMC551823

[B83] Li D. Gao X. Ma X. Wang M. Cheng C. Xue T. (2024). Aging-induced tRNAGlu-derived fragment impairs glutamate biosynthesis by targeting mitochondrial translation-dependent cristae organization. *Cell Metab.* 36 1059–1075.e9. 10.1016/j.cmet.2024.02.011 38458203

[B84] Li E. Yin H. Su M. Li Q. Zhao Y. Zhang L. (2023). Inhibition of ferroptosis alleviates chronic unpredictable mild stress-induced depression in mice via tsRNA-3029b. *Brain Res. Bull.* 204:110773. 10.1016/j.brainresbull.2023.110773 37793597

[B85] Li N. Yao S. Yu G. Lu L. Wang Z. (2024). tRFtarget 2.0: Expanding the targetome landscape of transfer RNA-derived fragments. *Nucleic Acids Res.* 52 D345–D350. 10.1093/nar/gkad815 37811890 PMC10767876

[B86] Li P.-F. Guo S.-C. Liu T. Cui H. Feng D. Yang A. (2020). Integrative analysis of transcriptomes highlights potential functions of transfer-RNA-derived small RNAs in experimental intracerebral hemorrhage. *Aging* 12 22794–22813. 10.18632/aging.103938 33203799 PMC7746353

[B87] Li Q. Hu B. Hu G.-W. Chen C.-Y. Niu X. Liu J. (2016). tRNA-derived small non-coding RNAs in response to ischemia inhibit angiogenesis. *Sci. Rep.* 6:20850. 10.1038/srep20850 26865164 PMC4749989

[B88] Li T. Zhen H. Wu W. Yang F. Cao Z. (2024). tsRNAs: A prospective, effective therapeutic intervention for neurodegenerative diseases. *CNS Neurosci. Ther.* 30:e70177. 10.1111/cns.70177 39690867 PMC11652788

[B89] Li Z. Stanton B. A. (2021). Transfer RNA-derived fragments, the underappreciated regulatory small RNAs in microbial pathogenesis. *Front. Microbiol.* 12:687632. 10.3389/fmicb.2021.687632 34079534 PMC8166272

[B90] Liu S. Chen Y. Ren Y. Zhou J. Ren J. Lee I. (2018). A tRNA-derived RNA fragment plays an important role in the mechanism of arsenite -induced cellular responses. *Sci. Rep.* 8:16838. 10.1038/s41598-018-34899-2 30442959 PMC6237853

[B91] Loher P. Londin E. Ilieva H. Pasinelli P. Rigoutsos I. (2025). Re-analyses of samples from amyotrophic lateral sclerosis patients and controls identify many novel small RNAs with diagnostic and prognostic potential. *Mol. Neurobiol*. 62 8135–8149. 10.1007/s12035-025-04747-2 39982687 PMC12208959

[B92] Lou Y.-L. Guo F. Liu F. Gao F.-L. Zhang P.-Q. Niu X. (2012). miR-210 activates notch signaling pathway in angiogenesis induced by cerebral ischemia. *Mol. Cell. Biochem.* 370 45–51. 10.1007/s11010-012-1396-6 22833359

[B93] Love M. I. Huber W. Anders S. (2014). Moderated estimation of fold change and dispersion for RNA-seq data with DESeq2. *Genome Biol.* 15:550. 10.1186/s13059-014-0550-8 25516281 PMC4302049

[B94] Lu H. Liu L. Han S. Wang B. Qin J. Bu K. (2021). Expression of tiRNA and tRF in APP/PS1 transgenic mice and the change of related proteins expression. *Ann. Transl. Med.* 9:1457. 10.21037/atm-21-4318 34734009 PMC8506760

[B95] Lv X. Zhang R. Li S. Jin X. (2024). tRNA modifications and dysregulation: Implications for brain diseases. *Brain Sci.* 14:633. 10.3390/brainsci14070633 39061374 PMC11274612

[B96] Lyons S. M. Achorn C. Kedersha N. L. Anderson P. J. Ivanov P. (2016). YB-1 regulates tiRNA-induced stress granule formation but not translational repression. *Nucleic Acids Res.* 44 6949–6960. 10.1093/nar/gkw418 27174937 PMC5001593

[B97] Lyons S. M. Fay M. M. Ivanov P. (2018). The role of RNA modifications in the regulation of tRNA cleavage. *FEBS Lett.* 592 2828–2844. 10.1002/1873-3468.13205 30058219 PMC6986807

[B98] Maciak K. Dziedzic A. Miller E. Saluk-Bijak J. (2021). miR-155 as an important regulator of multiple sclerosis pathogenesis. A review. *Int. J. Mol. Sci.* 22:4332. 10.3390/ijms22094332 33919306 PMC8122504

[B99] Madrer N. Vaknine-Treidel S. Zorbaz T. Tzur Y. Bennett E. R. Drori P. (2025). Pre-symptomatic Parkinson’s disease blood test quantifying repetitive sequence motifs in transfer RNA fragments. *Nat. Aging* 5 868–882. 10.1038/s43587-025-00851-z 40216989 PMC12092246

[B100] Magee R. Londin E. Rigoutsos I. (2019). TRNA-derived fragments as sex-dependent circulating candidate biomarkers for Parkinson’s disease. *Parkinsonism Relat. Disord.* 65 203–209. 10.1016/j.parkreldis.2019.05.035 31402278

[B101] Magen I. Yacovzada N. S. Yanowski E. Coenen-Stass A. Grosskreutz J. Lu C.-H. (2021). Circulating miR-181 is a prognostic biomarker for amyotrophic lateral sclerosis. *Nat. Neurosci.* 24 1534–1541. 10.1038/s41593-021-00936-z 34711961

[B102] Mahmood T. Yang P.-C. (2012). Western blot: Technique, theory, and trouble shooting. *N. Am. J. Med. Sci.* 4 429–434. 10.4103/1947-2714.100998 23050259 PMC3456489

[B103] Mann M. Wright P. R. Backofen R. (2017). IntaRNA 2.0: Enhanced and customizable prediction of RNA-RNA interactions. *Nucleic Acids Res.* 45 W435–W439. 10.1093/nar/gkx279 28472523 PMC5570192

[B104] Manu M. S. Hohjoh H. Yamamura T. (2021). Extracellular vesicles as pro- and anti-inflammatory mediators, biomarkers and potential therapeutic agents in multiple sclerosis. *Aging Dis.* 12 1451–1461. 10.14336/AD.2021.0513 34527421 PMC8407883

[B105] Marcelo A. Koppenol R. de Almeida L. P. Matos C. A. Nóbrega C. (2021). Stress granules, RNA-binding proteins and polyglutamine diseases: Too much aggregation? *Cell Death Dis.* 12 592. 10.1038/s41419-021-03873-8 34103467 PMC8187637

[B106] Martens-Uzunova E. S. Kusuma G. D. Crucitta S. Lim H. K. Cooper C. Riches J. E. (2021). Androgens alter the heterogeneity of small extracellular vesicles and the small RNA cargo in prostate cancer. *J. Extracell. Vesicles* 10:e12136. 10.1002/jev2.12136 34434533 PMC8374107

[B107] Martinez F. J. Lee J. H. Lee J. E. Blanco S. Nickerson E. Gabriel S. (2012). Whole exome sequencing identifies a splicing mutation in NSUN2 as a cause of a Dubowitz-like syndrome. *J. Med. Genet.* 49 380–385. 10.1136/jmedgenet-2011-100686 22577224 PMC4771841

[B108] Maute R. L. Schneider C. Sumazin P. Holmes A. Califano A. Basso K. (2013). tRNA-derived microRNA modulates proliferation and the DNA damage response and is down-regulated in B cell lymphoma. *Proc. Natl. Acad. Sci. U S A.* 110 1404–1409. 10.1073/pnas.1206761110 23297232 PMC3557069

[B109] McKinnon K. M. (2018). Flow cytometry: An overview. *Curr. Protoc. Immunol.* 120:51. 10.1002/cpim.40 29512141 PMC5939936

[B110] Miranda K. C. Huynh T. Tay Y. Ang Y.-S. Tam W.-L. Thomson A. M. (2006). A pattern-based method for the identification of MicroRNA binding sites and their corresponding heteroduplexes. *Cell* 126 1203–1217. 10.1016/j.cell.2006.07.031 16990141

[B111] Monahan P. E. Jooss K. Sands M. S. (2002). Safety of adeno-associated virus gene therapy vectors: A current evaluation. *Expert Opin. Drug Saf.* 1 79–91. 10.1517/14740338.1.1.79 12904163

[B112] Moore C. B. Guthrie E. H. Huang M. T.-H. Taxman D. J. (2010). Short hairpin RNA (shRNA): Design, delivery, and assessment of gene knockdown. *Methods Mol. Biol.* 629 141–158. 10.1007/978-1-60761-657-3_10 20387148 PMC3679364

[B113] Muneretto G. Plazzi F. Passamonti M. (2024). Mitochondrion-to-nucleus communication mediated by RNA export: A survey of potential mechanisms and players across eukaryotes. *Biol. Lett.* 20:20240147. 10.1098/rsbl.2024.0147 38982851 PMC11283861

[B114] Needhamsen M. Khoonsari P. E. Zheleznyakova G. Y. Piket E. Hagemann-Jensen M. Han Y. (2022). Integration of small RNAs from plasma and cerebrospinal fluid for classification of multiple sclerosis. *Front. Genet.* 13:1042483. 10.3389/fgene.2022.1042483 36468035 PMC9713411

[B115] Nersisyan S. Loher P. Nazeraj I. Shao Z. Fullard J. F. Voloudakis G. (2024). Comprehensive profiling of small RNAs and their changes and linkages to mRNAs in schizophrenia and bipolar disorder. *bioRxiv [Preprint]* 10.1101/2024.12.24.630254 39763727 PMC11703252

[B116] Nguyen T. T. M. van der Bent M. L. Wermer M. J. H. van den Wijngaard I. R. van Zwet E. W. de Groot B. (2020). Circulating tRNA Fragments as a novel biomarker class to distinguish acute stroke subtypes. *Int. J. Mol. Sci.* 22 :135. 10.3390/ijms22010135 33374482 PMC7796003

[B117] Nowak A. Wicik Z. Wolska M. Shahzadi A. Szwed P. Jarosz-Popek J. (2022). The role of non-coding RNAs in neuroinflammatory process in multiple sclerosis. *Mol. Neurobiol.* 59 4651–4668. 10.1007/s12035-022-02854-y 35589919

[B118] Oberbauer V. Drino A. Schaefer M. R. (2025). Determining small RNA-interacting proteomes using endogenously modified tRNA-derived RNAs. *Methods Enzymol.* 711 356–380. 10.1016/bs.mie.2024.11.005 39952715

[B119] Paldor I. Madrer N. Vaknine Treidel S. Shulman D. Greenberg D. S. Soreq H. (2023). Cerebrospinal fluid and blood profiles of transfer RNA fragments show age, sex, and Parkinson’s disease-related changes. *J. Neurochem.* 164 671–683. 10.1111/jnc.15723 36354307

[B120] Pan Q. Han T. Li G. (2021). Novel insights into the roles of tRNA-derived small RNAs. *RNA Biol.* 18 2157–2167. 10.1080/15476286.2021.1922009 33998370 PMC8632077

[B121] Pantano L. Friedländer M. R. Escaramís G. Lizano E. Pallarès-Albanell J. Ferrer I. (2016). Specific small-RNA signatures in the amygdala at premotor and motor stages of Parkinson’s disease revealed by deep sequencing analysis. *Bioinformatics* 32 673–681. 10.1093/bioinformatics/btv632 26530722

[B122] Pawar K. Kawamura T. Kirino Y. (2024). The tRNAVal half: A strong endogenous Toll-like receptor 7 ligand with a 5’-terminal universal sequence signature. *Proc. Natl. Acad. Sci. U S A.* 121:e2319569121. 10.1073/pnas.2319569121 38683985 PMC11087793

[B123] Pellegrini M. Bergonzoni G. Perrone F. Squitieri F. Biagioli M. (2022). Current diagnostic methods and non-coding RNAs as possible biomarkers in Huntington’s disease. *Genes* 13:2017. 10.3390/genes13112017 36360254 PMC9689996

[B124] Pliatsika V. Loher P. Magee R. Telonis A. G. Londin E. Shigematsu M. (2018). MINTbase v2.0: A comprehensive database for tRNA-derived fragments that includes nuclear and mitochondrial fragments from all The Cancer Genome Atlas projects. *Nucleic Acids Res.* 46 D152–D159. 10.1093/nar/gkx1075 29186503 PMC5753276

[B125] Prehn J. H. M. Jirström E. (2020). Angiogenin and tRNA fragments in Parkinson’s disease and neurodegeneration. *Acta Pharmacol. Sin.* 41 442–446. 10.1038/s41401-020-0375-9 32144338 PMC7470775

[B126] Puhakka N. Das Gupta S. Vuokila N. Pitkänen A. (2022). Transfer RNA-derived fragments and isomiRs are novel components of chronic TBI-induced neuropathology. *Biomedicines* 10:136. 10.3390/biomedicines10010136 35052815 PMC8773447

[B127] Qin C. Feng H. Zhang C. Zhang X. Liu Y. Yang D.-G. (2019). Differential expression profiles and functional prediction of tRNA-derived small RNAs in rats after traumatic spinal cord injury. *Front. Mol. Neurosci.* 12:326. 10.3389/fnmol.2019.00326 31998075 PMC6968126

[B128] Qin C. Xu P.-P. Zhang X. Zhang C. Liu C.-B. Yang D.-G. (2020). Pathological significance of tRNA-derived small RNAs in neurological disorders. *Neural Regen. Res.* 15 212–221. 10.4103/1673-5374.265560 31552886 PMC6905339

[B129] Qiu P. Jiang Q. Song H. (2024). Unveiling the hidden world of transfer RNA-derived small RNAs in inflammation. *J. Inflamm.* 21:46. 10.1186/s12950-024-00418-6 39533297 PMC11556027

[B130] Rashad S. Tominaga T. Niizuma K. (2021). The cell and stress-specific canonical and noncanonical tRNA cleavage. *J. Cell. Physiol.* 236 3710–3724. 10.1002/jcp.30107 33043995

[B131] Rawat P. Boehning M. Hummel B. Aprile-Garcia F. Pandit A. S. Eisenhardt N. (2021). Stress-induced nuclear condensation of NELF drives transcriptional downregulation. *Mol. Cell.* 81 1013–1026.e11. 10.1016/j.molcel.2021.01.016 33548202 PMC7939545

[B132] Ren J. Wu X. Shang F.-F. Qi Y. Tang Z. Wen C. (2022). The tRNA-Cys-GCA derived tsRNAs suppress tumor progression of gliomas via regulating VAV2. *Dis. Markers* 2022:8708312. 10.1155/2022/8708312 36426134 PMC9681550

[B133] Rojas P. Ramírez A. I. Fernández-Albarral J. A. López-Cuenca I. Salobrar-García E. Cadena M. (2020). Amyotrophic lateral sclerosis: A neurodegenerative motor neuron disease with ocular involvement. *Front. Neurosci.* 14:566858. 10.3389/fnins.2020.566858 33071739 PMC7544921

[B134] Roos R. A. C. (2010). Huntington’s disease: A clinical review. *Orphanet. J. Rare Dis.* 5:40. 10.1186/1750-1172-5-40 21171977 PMC3022767

[B135] Saikia M. Hatzoglou M. (2015). The many virtues of tRNA-derived stress-induced RNAs (tiRNAs): Discovering novel mechanisms of stress response and effect on human health. *J. Biol. Chem.* 290 29761–29768. 10.1074/jbc.R115.694661 26463210 PMC4705977

[B136] Sato K. Rashad S. Niizuma K. Tominaga T. (2020). Stress induced tRNA halves (tiRNAs) as biomarkers for stroke and stroke therapy; pre-clinical study. *Neuroscience* 434 44–54. 10.1016/j.neuroscience.2020.03.018 32200075

[B137] Schaffer A. E. Eggens V. R. C. Caglayan A. O. Reuter M. S. Scott E. Coufal N. G. (2014). CLP1 founder mutation links tRNA splicing and maturation to cerebellar development and neurodegeneration. *Cell* 157 651–663. 10.1016/j.cell.2014.03.049 24766810 PMC4128918

[B138] Schimmel P. (2018). The emerging complexity of the tRNA world: Mammalian tRNAs beyond protein synthesis. *Nat. Rev. Mol. Cell. Biol.* 19 45–58. 10.1038/nrm.2017.77 28875994

[B139] Sebastià J. Kieran D. Breen B. King M. A. Netteland D. F. Joyce D. (2009). Angiogenin protects motoneurons against hypoxic injury. *Cell. Death Differ.* 16 1238–1247. 10.1038/cdd.2009.52 19444281

[B140] Sharma U. Conine C. C. Shea J. M. Boskovic A. Derr A. G. Bing X. Y. (2016). Biogenesis and function of tRNA fragments during sperm maturation and fertilization in mammals. *Science* 351 391–396. 10.1126/science.aad6780 26721685 PMC4888079

[B141] Shulman D. Dubnov S. Zorbaz T. Madrer N. Paldor I. Bennett D. A. (2023). Sex-specific declines in cholinergic-targeting tRNA fragments in the nucleus accumbens in Alzheimer’s disease. *Alzheimers Dement.* 19 5159–5172. 10.1002/alz.13095 37158312 PMC10632545

[B142] Skorupa A. King M. A. Aparicio I. M. Dussmann H. Coughlan K. Breen B. (2012). Motoneurons secrete angiogenin to induce RNA cleavage in astroglia. *J. Neurosci.* 32 5024–5038. 10.1523/JNEUROSCI.6366-11.2012 22496549 PMC6622103

[B143] Soares A. R. Santos M. (2017). Discovery and function of transfer RNA-derived fragments and their role in disease. *Wiley Interdiscip. Rev. RNA* 8:1423. 10.1002/wrna.1423 28608481

[B144] Su Z. Frost E. L. Lammert C. R. Przanowska R. K. Lukens J. R. Dutta A. (2020a). tRNA-derived fragments and microRNAs in the maternal-fetal interface of a mouse maternal-immune-activation autism model. *RNA Biol.* 17 1183–1195. 10.1080/15476286.2020.1721047 31983265 PMC7549640

[B145] Su Z. Monshaugen I. Wilson B. Wang F. Klungland A. Ougland R. (2022). TRMT6/61A-dependent base methylation of tRNA-derived fragments regulates gene-silencing activity and the unfolded protein response in bladder cancer. *Nat. Commun.* 13:2165. 10.1038/s41467-022-29790-8 35444240 PMC9021294

[B146] Su Z. Wilson B. Kumar P. Dutta A. (2020b). Noncanonical roles of tRNAs: tRNA fragments and beyond. *Annu. Rev. Genet.* 54 47–69. 10.1146/annurev-genet-022620-101840 32841070 PMC7686126

[B147] Sun B. Chen Z. Chi Q. Zhang Y. Gao B. (2021). Endogenous tRNA-derived small RNA (tRF3-Thr-AGT) inhibits ZBP1/NLRP3 pathway-mediated cell pyroptosis to attenuate acute pancreatitis (AP). *J. Cell. Mol. Med.* 25 10441–10453. 10.1111/jcmm.16972 34643045 PMC8581331

[B148] Tahiri I. Llana S. R. Díaz-Castro F. Claret M. Obri A. (2025). AgRP neurons shape the sperm small RNA payload. *Sci. Rep.* 15:7206. 10.1038/s41598-025-91391-4 40021730 PMC11871312

[B149] Tan X. Liu Y. Liu Y. Zhang T. Cong S. (2021). Dysregulation of long non-coding RNAs and their mechanisms in Huntington’s disease. *J. Neurosci. Res.* 99 2074–2090. 10.1002/jnr.24825 34031910

[B150] Thompson D. M. Lu C. Green P. J. Parker R. (2008). tRNA cleavage is a conserved response to oxidative stress in eukaryotes. *RNA* 14 2095–2103. 10.1261/rna.1232808 18719243 PMC2553748

[B151] Tian H. Hu Z. Wang C. (2022). The therapeutic potential of tRNA-derived small RNAs in neurodegenerative disorders. *Aging Dis.* 13 389–401. 10.14336/AD.2021.0903 35371602 PMC8947841

[B152] Tian M. Liu X. Wang D. Wang Y. Wang S. Wei J. (2025). Differences of tsRNA expression profiles efficiently discriminate monozygotic twins in peripheral blood. *Forensic Sci. Int. Genet.* 77:103242. 10.1016/j.fsigen.2025.103242 39999615

[B153] Tomar A. Gomez-Velazquez M. Gerlini R. Comas-Armangué G. Makharadze L. Kolbe T. (2024). Epigenetic inheritance of diet-induced and sperm-borne mitochondrial RNAs. *Nature* 630 720–727. 10.1038/s41586-024-07472-3 38839949 PMC11186758

[B154] Tong L. Zhang W. Qu B. Zhang F. Wu Z. Shi J. (2020). The tRNA-derived fragment-3017A promotes metastasis by inhibiting NELL2 in human gastric cancer. *Front. Oncol.* 10:570916. 10.3389/fonc.2020.570916 33665159 PMC7921707

[B155] Torres A. G. Martí E. (2021). Toward an understanding of extracellular tRNA biology. *Front. Mol. Biosci.* 8:662620. 10.3389/fmolb.2021.662620 33937338 PMC8082309

[B156] Tripolszki K. Danis J. Padhi A. K. Gomes J. Bozó R. Nagy Z. F. (2019). Angiogenin mutations in Hungarian patients with amyotrophic lateral sclerosis: Clinical, genetic, computational, and functional analyses. *Brain Behav.* 9:e01293. 10.1002/brb3.1293 31025543 PMC6576160

[B157] Tu M. Zuo Z. Chen C. Zhang X. Wang S. Chen C. (2023). Transfer RNA-derived small RNAs (tsRNAs) sequencing revealed a differential expression landscape of tsRNAs between glioblastoma and low-grade glioma. *Gene* 855:147114. 10.1016/j.gene.2022.147114 36526122

[B158] van Es M. A. Schelhaas H. J. van Vught P. W. J. Ticozzi N. Andersen P. M. Groen E. J. N. (2011). Angiogenin variants in Parkinson disease and amyotrophic lateral sclerosis. *Ann. Neurol.* 70 964–973. 10.1002/ana.22611 22190368 PMC5560057

[B159] Van Haute L. Lee S.-Y. McCann B. J. Powell C. A. Bansal D. Vasiliauskaitė L. (2019). NSUN2 introduces 5-methylcytosines in mammalian mitochondrial tRNAs. *Nucleic Acids Res.* 47 8720–8733. 10.1093/nar/gkz559 31276587 PMC6822013

[B160] Wang B. Xia L. Zhu D. Zeng H. Wei B. Lu L. (2022). Paternal high-fat diet altered sperm 5’tsRNA-Gly-GCC is associated with enhanced gluconeogenesis in the offspring. *Front. Mol. Biosci.* 9:857875. 10.3389/fmolb.2022.857875 35480893 PMC9035875

[B161] Wang H. (2021). MicroRNAs, multiple sclerosis, and depression. *Int. J. Mol. Sci.* 22:7802. 10.3390/ijms22157802 34360568 PMC8346048

[B162] Wang X. Gao M. Song J. Li M. Chen Y. Lv Y. (2025). Differential expression of tRNA-derived small RNA markers of antidepressant response and functional forecast of duloxetine in MDD patients. *Genes* 16:162. 10.3390/genes16020162 40004491 PMC11855652

[B163] Wang Z. Xiang L. Shao J. Yuan Z. (2006). The 3’ CCACCA sequence of tRNAAla(UGC) is the motif that is important in inducing Th1-like immune response, and this motif can be recognized by Toll-like receptor 3. *Clin. Vaccine Immunol.* 13 733–739. 10.1128/CVI.00019-06 16829609 PMC1489575

[B164] Wei D. Zhai B. Zeng H. Liu L. Gao H. Xiang S. (2025). TRMT10A regulates tRNA-ArgCCT m1G9 modification to generate tRNA-derived fragments influencing vasculogenic mimicry formation in glioblastoma. *Cell Death Dis.* 16:209. 10.1038/s41419-025-07548-6 40140670 PMC11947273

[B165] Wei Y. Yang C.-R. Wei Y.-P. Zhao Z.-A. Hou Y. Schatten H. (2014). Paternally induced transgenerational inheritance of susceptibility to diabetes in mammals. *Proc. Natl. Acad. Sci. U S A.* 111 1873–1878. 10.1073/pnas.1321195111 24449870 PMC3918818

[B166] Weng Q. Wang Y. Xie Y. Yu X. Zhang S. Ge J. (2022). Extracellular vesicles-associated tRNA-derived fragments (tRFs): Biogenesis, biological functions, and their role as potential biomarkers in human diseases. *J. Mol. Med.* 100 679–695. 10.1007/s00109-022-02189-0 35322869 PMC9110440

[B167] Wilson B. Dutta A. (2022). Function and therapeutic implications of tRNA derived small RNAs. *Front. Mol. Biosci.* 9:888424. 10.3389/fmolb.2022.888424 35495621 PMC9043108

[B168] Winek K. Lobentanzer S. Nadorp B. Dubnov S. Dames C. Jagdmann S. (2020). Transfer RNA fragments replace microRNA regulators of the cholinergic poststroke immune blockade. *Proc. Natl. Acad. Sci. U S A.* 117 32606–32616. 10.1073/pnas.2013542117 33288717 PMC7768686

[B169] Witzel S. Mayer K. Oeckl P. (2022). Biomarkers for amyotrophic lateral sclerosis. *Curr. Opin. Neurol.* 35 699–704. 10.1097/WCO.0000000000001094 35942674

[B170] Wu W. Lee I. Spratt H. Fang X. Bao X. (2021). tRNA-Derived fragments in Alzheimer’s disease: Implications for new disease biomarkers and neuropathological mechanisms. *J. Alzheimers Dis.* 79 793–806. 10.3233/JAD-200917 33337366 PMC8485948

[B171] Wu W. Shen A. Lee I. Miranda-Morales E. G. Spratt H. Pappolla M. A. (2023). Changes of tRNA-derived fragments by Alzheimer’s disease in cerebrospinal fluid and blood serum. *J. Alzheimers Dis.* 96 1285–1304. 10.3233/JAD-230412 37980659 PMC10832608

[B172] Wu Y. E. Parikshak N. N. Belgard T. G. Geschwind D. H. (2016). Genome-wide, integrative analysis implicates microRNA dysregulation in autism spectrum disorder. *Nat. Neurosci.* 19 1463–1476. 10.1038/nn.4373 27571009 PMC5841760

[B173] Xie Y. Yao L. Yu X. Ruan Y. Li Z. Guo J. (2020). Action mechanisms and research methods of tRNA-derived small RNAs. *Signal. Transduct. Target. Ther.* 5:109. 10.1038/s41392-020-00217-4 32606362 PMC7326991

[B174] Xu T. Yuan J. Song F. Zhang N. Gao C. Chen Z. (2024). Exploring the functional role of tRF-39-8HM2OSRNLNKSEKH9 in hepatocellular carcinoma. *Heliyon* 10:e27153. 10.1016/j.heliyon.2024.e27153 38455567 PMC10918225

[B175] Xu X.-J. Yang M.-S. Zhang B. Ge Q.-Q. Niu F. Dong J.-Q. (2022). Genome-wide interrogation of transfer RNA-derived small RNAs in a mouse model of traumatic brain injury. *Neural Regen. Res.* 17 386–394. 10.4103/1673-5374.314315 34269214 PMC8463968

[B176] Yamasaki S. Ivanov P. Hu G.-F. Anderson P. (2009). Angiogenin cleaves tRNA and promotes stress-induced translational repression. *J. Cell. Biol.* 185 35–42. 10.1083/jcb.200811106 19332886 PMC2700517

[B177] Yang M. Mo Y. Ren D. Liu S. Zeng Z. Xiong W. (2023). Transfer RNA-derived small RNAs in tumor microenvironment. *Mol. Cancer* 22:32. 10.1186/s12943-023-01742-w 36797764 PMC9933334

[B178] Yang Z.-Y. Tang T. Li P.-F. Li X.-X. Wu Y. Feng D.-D. (2022). Systematic analysis of tRNA-derived small RNAs reveals therapeutic targets of Xuefu Zhuyu decoction in the cortexes of experimental traumatic brain injury. *Phytomedicine* 102:154168. 10.1016/j.phymed.2022.154168 35623157

[B179] Yasinjan F. Xing Y. Geng H. Guo R. Yang L. Liu Z. (2023). Immunotherapy: A promising approach for glioma treatment. *Front. Immunol.* 14:1255611. 10.3389/fimmu.2023.1255611 37744349 PMC10512462

[B180] Ye L. Dittlau K. S. Sicart A. Janky R. Van Damme P. Van Den Bosch L. (2025). Sporadic ALS hiPSC-derived motor neurons show axonal defects linked to altered axon guidance pathways. *Neurobiol. Dis.* 206:106815. 10.1016/j.nbd.2025.106815 39884586

[B181] Yousuf A. Qurashi A. (2021). Non-coding RNAs in the pathogenesis of multiple sclerosis. *Front. Genet.* 12:717922. 10.3389/fgene.2021.717922 34659340 PMC8514772

[B182] Yu J. Zhang X. Cai C. Zhou T. Chen Q. (2025). Small RNA and toll-like receptor interactions: Origins and disease mechanisms. *Trends Biochem. Sci.* 50 385–401. 10.1016/j.tibs.2025.01.004 39956743 PMC12048287

[B183] Yu M. Liu P. Lu Y. (2024). Protocol for detection of tRNA-derived fragments in cells, tissues, and plasma. *STAR Protoc.* 5:103435. 10.1016/j.xpro.2024.103435 39514391 PMC11574804

[B184] Yuan J. Song Z. Liu J. Rahman K. U. Zhou Q. Liu G. (2024). Transfer RNAs and transfer RNA-derived small RNAs in cerebrovascular diseases. *Exp. Neurol.* 382:114971. 10.1016/j.expneurol.2024.114971 39326819

[B185] Yuan Y. Li J. He Z. Fan X. Mao X. Yang M. (2021). tRNA-derived fragments as new hallmarks of aging and age-related diseases. *Aging Dis.* 12 1304–1322. 10.14336/AD.2021.0115 34341710 PMC8279533

[B186] Zhang L. Liu J. Hou Y. (2023). Classification, function, and advances in tsRNA in non-neoplastic diseases. *Cell Death Dis.* 14:748. 10.1038/s41419-023-06250-9 37973899 PMC10654580

[B187] Zhang S. Li H. Zheng L. Li H. Feng C. Zhang W. (2019). Identification of functional tRNA-derived fragments in senescence-accelerated mouse prone 8 brain. *Aging* 11 10485–10498. 10.18632/aging.102471 31746776 PMC6914438

[B188] Zhang X. Shi J. Thakore P. Gonzales A. L. Earley S. Chen Q. (2024). Mitochondrial small RNA alterations associated with increased lysosome activity in an Alzheimer’s disease mouse model uncovered by PANDORA-seq. *bioRxiv [Preprint]* 10.1101/2024.10.18.619155 40243634 PMC11988842

[B189] Zhang X. Shi J. Thakore P. Gonzales A. L. Earley S. Chen Q. (2025). Mitochondrial small RNA alterations associated with increased lysosome activity in an Alzheimer’s disease mouse model uncovered by PANDORA-seq. *Int. J. Mol. Sci.* 26:3019. 10.3390/ijms2607301 40243634 PMC11988842

[B190] Zhang X. Trebak F. Souza L. A. C. Shi J. Zhou T. Kehoe P. G. (2020). Small RNA modifications in Alzheimer’s disease. *Neurobiol. Dis.* 145:105058. 10.1016/j.nbd.2020.105058 32835860 PMC7572745

[B191] Zhang Y. Ren L. Sun X. Zhang Z. Liu J. Xin Y. (2021). Angiogenin mediates paternal inflammation-induced metabolic disorders in offspring through sperm tsRNAs. *Nat. Commun.* 12:6673. 10.1038/s41467-021-26909-1 34845238 PMC8630171

[B192] Zhang Z.-Y. Zhang C.-H. Yang J.-J. Xu P.-P. Yi P.-J. Hu M.-L. (2021). Genome-wide analysis of hippocampal transfer RNA-derived small RNAs identifies new potential therapeutic targets of Bushen Tiansui formula against Alzheimer’s disease. *J. Integr. Med.* 19 135–143. 10.1016/j.joim.2020.12.005 33334712

[B193] Zhao S. Wang Y. Zhou L. Li Z. Weng Q. (2025). Exploring the potential of tsRNA as biomarkers for diagnosis and treatment of neurogenetic disorders. *Mol. Neurobiol*. 62 8518–8539. 10.1007/s12035-025-04760-5 40009263

