# Supplementary material for: Deciphering the role of tRNA-derived fragments in neurological and psychiatric disease pathogenesis
Source: Front Cell Neurosci. 2025 Dec 3;19:1663788. doi: 10.3389/fncel.2025.1663788 (PMC12708565; doi:10.3389/fncel.2025.1663788)
Supplement: Supplementary file 1 [file Table_1.docx]

**Supplementary Table 1.** Cell types, study models and results of the exhibited in vitro studies

| CELL TYPE | MODEL | RESULT | PMID ID |
| --- | --- | --- | --- |
| Neuroblastoma cells (SH-SY5Y cell line) | 5′ Tyr-tRF and 3′ Tyr-tRF as well as control-tRF (5′ arginine-tRF [5′ Arg-tRF]) transfected cell line | Accumulated 5′ Tyr-tRFs impair neural differentiation by promoting cell death in human neuroblastoma cells. | 32143824 |
| Motoneuronal cells (NSC34 cell line) | Cells transiently transfected with pIRES2-DsRed2 constructs carrying ANG mutants (K40I, R31K, K17I, Q12L, I46V, or C39W) | Neuroprotective effect is lost under hypoxia in ALS-associated ANG mutations, including K40I, Q12L, K17I, R31K, C39W, and I46V | 19444281 |
| Neuronal cells (P19 cell line) | ANG-NCI 65828 inhibitor treated cells | ANG play a role in neurite pathfinding | 17468498 |
| Glioblastoma cells (MZ-294 cell line) | Recombinant human angiogenin treated cells | 5′ValCAC tRNA fragment might hold biomarker potential in ALS | 33543130 |
| Rat neuronal cells (PC12 cell line) | H_2_O_2_ and arsenite exposure for oxidative stress induction in the cells | Angiogenin-mediated tiRNA generation can be induced in neuronal cells (PC12) by different cell stressors, including ischemia-reperfusion, arsenite and hydrogen peroxide. | 29431851 |
| Mouse primary cells (GBM cells) | EGFR- and PDGFRA-driven mouse primary GBM and primary astrocytes isolated from constitutive Cdkn2a null and conditional p53^2lox^ mice | The presence of tRFs in microvesicles and exosomes. | 31242427 |

**Supplementary Table 2.** Cell types, study models and results of the exhibited in vivo studies

| DISEASE | SPECIES | MODEL | SAMPLE | METHOD | RESULT | PMID ID |
| --- | --- | --- | --- | --- | --- | --- |
| NSUN2 gene mutation (Dubowitz-like syndrome) | **Mouse** | NSun2 -/- mice | Skin isolated 3.5-week-old male mice, frontal lobe of E18.5 embryonic mouse brains | tRNA -seq qPCR  Northern Blot | NSun2 loss induces angiogenin-dependent tRNA cleavage, causing 5′ tRF accumulation, translation disruption, neuronal death, and a phenotype reversible by angiogenin inhibition | 25063673 |
| Neuronal Nsun2 deficiency | **Mouse** | Nsun2 conditional knockout mice | Brain sample | RNA seq | NSun2 loss impairs neuronal differentiation and synaptic function, with effects worsened by angiogenin exposure | 34389722 |
| AD | **Mouse** | APP/PS1 transgenic mice | Mice hippocampus | tRF-seq qPCR | tRFs (tRF-Thr-CGT-003 and tRF-Leu-CAA-004) are differentially expressed | 34734009 |
| AD | **Mouse** | 5XFAD mice | Mice prefrontal brain tissues | Small RNA seq Pandora seq | According to pandora seq, 187 mitochondrial tsRNAs downregulated in the prefrontal cortex of the 5XFAD mouse brain.  mt-tsRNA-Gln, mt-tsRNA-His, and mt-tsRNA-Leu are reduced in AD cortex. | 40243634 |
| AD | **Rat** | Rat AD model (oligomeric Aβ1-42 given to generate AD model) | Rat hippocampus tissue | tsRNA-sequencing | 13 significantly dysregulated tsRNAs in AD rats compared with sham control rats | 33334712 |
| ALS | **Mouse** | mouse, TG SOD1G93A mice | Spinal cord and serum | small RNA-seq Northern Blot qPCR | **1**.5′ValCAC was elevated at disease onset in the spinal cord from slow-progressing SOD1G93A transgenic (TG) mice (ALS) **2**. Serum 5′ValCAC levels as a prognostic biomarker for ALS patients | 33543130 |
| ALS | **Mouse** | TDP-43A315T hemizygous mice, SOD1G93A mice, The FUS (1–359) mice | mouse primary cortical neurons | RNA seq | Neuronal stress responses by a specific tiRNA, 5’tiRNAGly-GCC, which upregulated in primary neurons exposed to ALS-relevant stresses and in the spinal cord of three ALS mouse models. | 39719207 |
| ALS,FTD and PD | **Mouse** | SOD1G93A and TDP43A315T mouse models of ALS, the TauP301S model of FTD, the parkin/POLG model of PD | ALS (spinal cord) FTD (hippocampus) PD (s. nigra) | Small RNA-seq qPCR | **1.** Higher expression of 5′ tiRNAs selectively in the two ALS models **2.** Lower expression of 3′ tRFs in both the ALS and FTD mouse models  **3.** Lower expression of itRF Arg in the PD model. | 39552337 |
| Ischemia | **Rat** | Rat brain ischemic model (carotid arteries with 4-0 nylon intraluminal suture) | Rat brain tissue | Small RNA-seq | tRNAVal- and tRNAGly-derived small RNAs account for the most abundant tRFs | 26865164 |
| Ischemia | **Drosophila and mice** | Mice ishemia mode- Distal middle cerebral artery occlusion (MCAO) model | Primary mouse neurons | tRF seq qPCR | Transfected tRFs induced neuron swelling and death. The most affected pathway mitochondrial metabolism. | 34462418 |
| Stroke | **Rat** | Rat model of focal cerebral ischemia–reperfusion (I/R) injury(carotid bifurcation exposed, external carotid artery ligated) | Rat brain tissue | Nothern blot qPCR | tiRNAs were induced after I/R and Minocycline therapy reduced global tiRNA levels. | 32200075 |
| MDD | **Mouse** | mice CUMS model | Sperm | Small RNA-seq | There were 26 up- and 988 down-regulated tsRNAs in the depression-like behavior group compared with those in the control group.  The down-regulated tsRNAs of tsRNA-Leu (149) and tsRNA-Gly (148) reached their peak in number | 39961446 |
| Depression | **Mouse** | mice CUMS model | mouse hippocampus | Small RNA-seq | **1**. 5'-tsRNAs were down-regulated in the CUMS group compared to the control group **2**. CUMS-induced mice treated with Fer-1, a total of 22 tsRNAs exhibited differential expression | 37793597 |
| ICH | **Rat** | Rat Intracerebral hemorrhage model(collagenase induced ICH model) | Rat brain tissue | Small RNA-seq | A total of 331 tsRNAs were identified (308 in sham and 309 in intracerebral hemorrhage). Among them, the validation revealed that 7 tsRNAs (1 up-regulated and 6 down-regulated) were significantly changed. | 33203799 |
| TBI | **Mouse** | Mouse traumatic brain injury model (controlled cortical impact model) | Cortex | tsRNA seq | 103 tsRNAs were differentially expressed after 72 hours of injury | 34269214 |
| TBI | **Rat** | Rat traumatic brain injury model (lateral fluid-percussion injury to induce TBI) | Ipsilateral thalamus and perilesional cortex | Small RNA-seq | 3'tRF-IleAAT and 3'tRF-LysTTT upregulated tRFs | 35052815 |
| TBI | **Rat** | Rat traumatic brain injury mode (controlled cortical impact model) | Cortex | tsRNA seq qRT-PCR | The expression of 732 tsRNAs was dysregulated | 35623157 |
| TBI | **Rat** | Rat traumatic brain injury model (controlled cortical impact) | Hippocampal tissues surrounding the hemorrhagic region | tsRNA seq qRT-PCR | 322 tsRNAs were altered comparing the TBI group with the sham group | 36164400 |
| SCI | **Rat** | SCI model | Spinal cord | Small RNA-seq qPCR | **1**. 47 tsRNAs specifically expressed in the sham group, and 28 tsRNAs specifically expressed in the SCI group. **2**. tRF-5 s was increased in SCI group **3**. BDNF expression was inhibited by the tiRNA-Gly-GCC-001 directly targeting its 3′UTR | 31998075 |

*AD: Alzheimer’s Disease, ALS: Amyotrophic Lateral Sclerosis, PD: Parkinson’s Disease, FTD: Frontotemporal Dementia, HD: Huntington’s Disease, MDD: Major Depressive Disorder, ICH: Intracerebral hemorrhage, TBI: Traumatic Brain Injury, SCZ: Schizophrenia

**Supplementary Table 3.** Cell types, study models and results of the exhibited clinic studies

| DISEASE | NUMBERS OF PARTICIPANTS | SAMPLE | METHOD | RESULT | PMID ID |
| --- | --- | --- | --- | --- | --- |
| Pontocerebellar hypoplasia | Detailed information was not provided | Archived umblical cord | DNA-seq | Supporting this, CLP1 mutant patient-derived neurons showed reduced mature tRNAs and increased intron-containing pre-tRNAs. | 24766810 |
| AD | 198 familial and 803 sporadic AD and 1010 control | Blood samples | DNA-seq | A novel nonsense ANG mutation (K73X) found in two AD patients results in a 51-amino acid truncation and loss of the catalytic site | 30188356 |
| AD | 13 control, 15 AD patients and 6 control, 6 AD patients (previous RNA seq) | Post-mortem human hippocampus samples | Northern Blot qPCR Re-analyzed short RNA-sequencing data | tRF5-GlyGCC and tRF5-GluCTC were significantly increased in the AD | 33337366 |
| AD | **CSF:** 24 AD, 8 MCI, 9 FTD, 17 control **Hippocampus:** 10 control, 13 AD **Serum:** 62 controls, 47 AD, and 11 MCI. | CSF, serum hippocampus | T4 PNK-RNA-seq qPCR | tRF5-ProAGG showed the potential as an AD biomarker and may play a role in disease progression. | 37980659 |
| AD | controls with no cognitive impairment (35 females, 30 males) and persons diagnosed with AD (28 females and 19 males) | Post-mortem samples from Nac, STG and hypothalamus | scRNA seq | **1.** In females, 10 tRFs identified with altered levels **2.** tRFs decline is more pronounced in the Nac than in other brain regions | 37158312 |
| AD | 16 live human brain samples (6 females, 10 males; 42-75 years old) | Brain tissue | Small RNA-seq Pull down assay | tDR-36:74-Asn-GTT-2-M2 upregulated in the CSF of female AD patients | 38862813 |
| PD | **CSF:** control (53), PD (46), AD (53)   **S. nigra:** 8 PD  475 PPMI (blood) and the 152 NIH (CSF) and 20 from GSE23676 (blood) previous RNA seq | Serum, plasma, CSF, brain samples | Small RNA seq qPCR  Re-analyzed short RNA-sequencing data | PD-specific transfer RNA fragments carrying a conserved sequence motif (RGTTCRA-tRFs) in the substantia nigra, cerebrospinal fluid and blood of patients with PD. Reduced MT-tRFs are present in PD but not in AD. | 40216989 |
| PD | 7 technical control and 21 human brain samples ( 7 premotor cases, 7 motor cases and 7 control) | Post-mortem brain tissue | Small RNA-seq | tRNA-ARG-CCG, tRNA-ARG-CCT and tRNAARG-CCG clusters identified | 26530722 |
| PD | 29 PD 33 control 67 PD 69 control 61 PD 71 control | Prefrontal cortex, CSF, and serum | Re-analyzed short RNA-seq data | **1.** 62 differently expressed (DE) tRF, are common to both CSF and prefrontal cortex **2.** tRF between PND and PDD in CSF and in serum shows sex-dependence | 31402278 |
| PD | **CSF:** 60 PD, 63 control  **Blood:** 360 PD, 183 control | Post-mortem CSF samples and blood samples | Re-analyzed short RNA-seq data | **1.** Long tRFs are expressed in higher levels in the CSF than in the blood.  **2.** The CSF showed a pronounced age-associated decline in the level of 3’-tRFs, i-tRFs **3.** More pronounced profile differences than the blood profiles between the sexes. | 36354307 |
| PD | Peripheral blood EVs (n = 6) | EVs from Serum samples of PD | tsRNA seq qPCR | 122 tRFs exhibiting upregulation and 69 tRFs exhibiting downregulation in PD-EVs. | 40254704 |
| ALS and PD | 6,471 ALS patients and 7,668 controls from 15 centers (13 from Europe and 2 from the USA) and PD patients from 6 centers (5 from Europe and 1 from the USA). | Brain samples | DNA-seq | ANG variants have been detected in both ALS and PD patients compared to control subjects | 22190368 |
| ALS | 136 sporadic ALS patients and 112 controls | Blood samples | DNA-seq | RW33, V103I and M-24I mutations were detected only in ALS patients | 31025543 |
| HD | 37 HD (19 used for sequencing) | Frozen samples (putamen, frontal cortex, and cerebellum) | Small RNA-seq qPCR | **1.** High levels of tRNA fragments (tRFs) in Huntigton putamen **2.** tRF derived from an Alanine tRNA may contribute to HD pathophysiology. | 33547932 |
| MS | (nRRMS = 20, nSPMS = 5 (+1), nCIS = 1, nRIS = 1) and neurological controls (nNINDC = 9, nINDC = 5) | Blood and CSF | Small RNA-seq | 3′-tRFs from plasma have major contribution to the RRMS classification | 36468035 |
| Acute Stroke | 75 patients with ischemic and 66 with hemorrhagic stroke) and 22 controls | Plasma | ELISA kit using the anti-m1A antibody | The plasma tRNA derivative level was significantly increased in both ischemic and hemorrhagic stroke patients compared to the healthy volunteers | 33207351 |
| Stroke | Samples from patients with modified Rankin Scale (mRS) values of 3 and below at discharge from the hospital, 240 cases | Blood samples | Small RNA-seq | Six up-regulated tRFs in the cohort of stroke patients | 33288717 |
| Acute ischemic stroke subtypes | 9 ischemic stroke, 8 intracerebral hemorrhage and 9 stroke mimics  20 ischemic stroke, 20 controls (previous RNA seq) | Plasma | small RNA-sequencing qPCR  Re-analyzed short RNA-sequencing data | Identified tRNA-derived fragments as a promising novel class of biomarkers to distinguish between acute IS, ICH and SM, HC. | 33374482 |
| Acute ischemic stroke | 17 acute ischemic stroke | Plasma | ELISA kit using the anti-m1A antibody | The plasma tRNA derivatives concentrations on admission increased, corresponding to the infarction size (r = 0.492, p = 0.038), and were associated with clinical outcomes | 35802994 |
| Epilepsy | 16 controls and pre-seizure or post-seizure samples from 16 patients with focal epilepsy | Blood samples | Small RNA-seq Biotin-labeled tRNA fragments pull-down assay | Biomarker for epilepsy patients | 31039137 |
| Glioma and GBM | GBMs (4 cases) and low-grade gliomas (5 cases) | GBMs and low-grade gliomas | RNA-seq qPCR | A total of 9 tsRNAs were selected as candidate tsRNAs according to the tsRNA expression level, among which 6 tsRNAs were highly expressed in GBMs and 3 tsRNAs were low expressed in GBMs | 36526122 |
| Glioma | 40 glioma tissues 12 non-tumor brain tissues | Glioma tissues and non-tumor brain tissues | qPCR Re-analyzed short RNA-seq data | **1.** 9 tsRNAs derived from tRNA-Cys-GCA, in the sncRNA-sequence data of glioma tissues  **2.** Several tsRNAs (ts-55, ts-60, and tRFdb-3003a/b) were remarkably down-regulated in gliomas | 36426134 |
| GBM | Detailed information was not provided | Glioma tissue specimens | tRF seq qPCR | GBM patients with low TRMT10A expression have poorer prognoses. | 40140670 |
| SCZ and BD | prefrontal cortex of 93 schizophrenia or bipolar disorder and 77 controls. | Post-mortem brain samples | SncRNA-seq | Two groups of trfs are upregulated in SCZ (5´-tRNA halves and tRFs with non-templated 3´ guanylation) | 39763727 |
| MDD | 258 patients (males 80; females 178) | Peripheral blood samples | Re-analyzed short RNA-sequencing data RNA seq | 10 tsRNAs significantly changed in the duloxetine response group after an 8-week therapy. | 40004491 |

*AD: Alzheimer’s Disease, PD: Parkinson’s Disease, ALS: Amyotrophic Lateral Sclerosis, HD: Huntington’s Disease, MS: Multiple Sclerosis, GBM: Glioblastoma, SCZ: Schizophrenia, BD: Bipolar Disorder, MDD: Major Depressive Disorder
